# Asthma and the risk of gastrointestinal disorders: a Mendelian randomization study

**DOI:** 10.1186/s12916-022-02283-7

**Published:** 2022-03-16

**Authors:** Dennis Freuer, Jakob Linseisen, Christa Meisinger

**Affiliations:** 1 University of Augsburg, University Hospital Augsburg, Stenglinstr. 2, 86156 Augsburg, Germany; 2 Institute for Medical Information Processing, Biometry, and Epidemiology, Ludwig-Maximilians-Universität München, Munich, Germany; 3 Independent Research Group Clinical Epidemiology, Helmholtz Zentrum München, German Research Center for Environmental Health, Munich, Germany

**Keywords:** Asthma, Gastrointestinal disorders, Mendelian randomization, Crohn’s disease, Ulcerative colitis, Peptic ulcer disease, Gastroesophageal reflux disease, Irritable bowel syndrome, Inflammatory bowel disease

## Abstract

**Background:**

The question of whether asthma is causally related to gastrointestinal disorders remained unanswered so far. Thus, this study investigated whether there is such a relation and whether the time of onset of asthma plays a role in the occurrence of the following gastrointestinal disorders: peptic ulcer disease (PUD), gastroesophageal reflux disease (GORD), irritable bowel syndrome (IBS), and inflammatory bowel disease (IBD) including the distinction between Crohn’s disease (CD) and ulcerative colitis (UC).

**Methods:**

Using summary data of genome-wide association studies (GWASs), we ran Mendelian randomization analyses based on up to 456,327 European participants. Outlier assessment, a series of sensitivity analyses and validation of IBD results in a second GWAS were performed to confirm the results.

**Results:**

Presented ORs represent the average change in the outcome per 2.72-fold increase in the prevalence of the exposure. Genetically predicted childhood-onset asthma was positively associated with PUD, GORD, and IBS with similar odds ratios near 1.003 and adjusted *P*-values from 0.007 (GORD) to 0.047 (PUD). Furthermore, it was inversely related to IBD (OR = 0.992, 95% CI: 0.986, 0.998, adjusted *P* = 0.023) and suggestively associated with its UC subtype (OR = 0.990, 95% CI: 0.982, 0.998, adjusted *P* = 0.059). There were no associations between genetically predicted adult-onset asthma and the mentioned gastrointestinal disorders.

**Conclusions:**

This study provides evidence that the presence of asthma onset in childhood increases the risk for GORD, PUD, and IBS but decreases the risk for IBD in adults. The lower risk for IBD may be attributed to a lower risk primarily for UC.

**Supplementary Information:**

The online version contains supplementary material available at 10.1186/s12916-022-02283-7.

## Background

Evidence from observational studies suggests a complex interplay between chronic lung disorders (e.g., asthma, chronic obstructive pulmonary disease, cystic fibrosis) and gastrointestinal diseases [1–3]. Furthermore, studies reported the occurrence of intestinal symptoms as part of a viral respiratory infection [4, 5]. This indicates that there is a cross-talk between these organ systems, termed as “gut-lung axis” [6]. A number of exposures in early-life have been linked to both the predisposition towards respiratory diseases and changes in the intestinal microbiota. However, clear mechanisms responsible for the intestinal-pulmonary cross-talk are not yet clear [7].

The most prevalent chronic respiratory disease in children and adults is asthma [8]. Several observational studies for example focused on the relationship between asthma and inflammatory bowel disease (IBD) [9], asthma and irritable bowel syndrome (IBS) [10], and asthma and gastroesophageal reflux disease (GORD) [11]. It was found that people with asthma are three times more likely to have gastroesophageal reflux than healthy people [11]. Additionally, it could be shown that the risk of asthma in subjects with IBS is twice the risk in persons without the disease [10]. However, so far, it is not clear which disease causes the other. This largely unclear relationship could not be clarified even in systematic reviews and meta-analyses [9, 10, 12].

This study aims to close this gap and is therefore dedicated to the causal effects of both childhood- and adult-onset asthma on peptic ulcer disease (PUD), GORD, IBS, and IBD.

## Methods

### Study design and population

Since observational studies are prone to reverse causation and unmeasured confounding, we performed a Mendelian randomization (MR) analysis to investigate causal effects of asthma and the time of onset on the mentioned gastrointestinal disorders [13]. The MR approach uses genetic variants, which are randomly allocated at meiosis and therefore independent of potential confounders biasing observational studies, as proxies for a risk factor in an instrumental variable analysis. Beyond that, to be considered as a valid instrument, a genetic variant must be strongly associated with the risk factor of interest and not affect the outcome directly, but only through exposure. For each genetic instrument, a causal estimate (known as Wald ratio) is calculated by dividing the variant-outcome by the variant-exposure association [14]. Subsequently, all Wald ratios are combined into an overall estimate using meta-analysis tools.

In a nested 2-sample MR within one study cohort, we considered summary statistics of GWASs including 314,633, 327,253, and 456,327 UK Biobank (UKB) participants with European ancestry for childhood-onset asthma, adult-onset asthma, and gastrointestinal disorders, respectively. In the following, we will refer to this as one-sample MR. Overall 13,962 asthma-diagnosed (main and secondary ICD10) cases between 0 and 19 years of age and 26,582 cases between 20 and 60 years of age were compared to the control group of 300,671 individuals without any allergic disease, such as asthma and hay fever [15] (Table 1). Regarding the gastrointestinal disorders, out of 456,327 subjects, 3.7% were diagnosed with PUD, 12% with GORD, 6.5% with IBS, and 1.5% with IBD [16] (Table 2).

**Table 1:** Description of the UKB exposures [15]. In both cases, the control group contains 300,671 individuals without any allergic disease

	Childhood-onset asthma	Adult-onset asthma
Considered age range for onset	0 to 19	20 to 60
Sample size	314,633	327,253
Cases, abs (rel)	13,962 (0.044)	26,582 (0.081)
Associated independent SNPs	102	47
Instrumental SNPs/instrumental SNPs in validation analyses	89/76	42/38

*Abbreviations*: *SNPs* single nucleotide polymorphisms

**Table 2:** Description of the outcomes according to Wu et al. [16]

	PUD	GORD	IBS	IBD
Sample size	456,327	456,327	456,327	456,327
Cases, abs (rel)	16,666 (0.037)	54,854 (0.120)	29,524 (0.065)	7045 (0.015)

*Abbreviations*: *PUD* peptic ulcer disease, *GORD* gastroesophageal reflux disease, *IBS* irritable bowel syndrome, *IBD* inflammatory bowel disease

Additionally, a two-sample MR validation analysis was performed with IBD as outcome using GWAS summary data of 86,640 European individuals from the International Inflammatory Bowel Disease Genetics (IIBDG) consortium (Table 3). Beyond that, these data allowed us to investigate the outcomes CD and ulcerative colitis UC as subtypes of IBD with 20,550 and 17,647 cases vs. 41,642 and 47,179 controls, respectively [17, 18].

**Table 3:** Description of the outcome IBD and its subtypes Crohn’s disease and ulcerative colitis according to Liu et al. [17] used in validation analyses

	IBD	Crohn’s disease	Ulcerative colitis
Sample size	86,640	62,192	64,826
Cases, abs (rel)	38,155 (0.44)	20,550 (0.33)	17,647 (0.27)

*Abbreviations*: *IBD* inflammatory bowel disease

As possible instruments, we selected single nucleotide polymorphisms (SNPs) with an imputation score ≥0.8 that were strongly associated with asthma based on the genome-wide significance threshold of *P* = 5 · 10^−8^. After the linkage disequilibrium clumping with a stringent cut-off *r*^2^ = 0.001, we harmonized the respective exposure and outcome datasets using effect allele frequencies, while removing palindromic SNPs with intermediate allele frequencies (i.e., minor allele frequency >0.42).

Thus, 89 childhood-onset and 42 adult-onset asthma associated SNPs remained as possible independent instruments for the one-sample MR analyses (Additional file 1: Tables S1 and S2). In the two-sample setting 76 potential genetic instruments were used for childhood-onset and 38 for adult-onset asthma, respectively (Additional file 1: Tables S3 and S4).

### Statistical analyses

Assessing heterogeneity is an important part of a MR study to identify invalid instruments. Wald ratios are assumed to estimate the same causal effect. Thus, heterogeneous SNPs that differ significantly from other Wald ratios indicate instruments that influence the outcome through an additional path (i.e., directly or through a confounder of the exposure-outcome association) rather than only through the exposure (known as horizontal pleiotropy). As a result, the estimates will be biased especially in presence of unbalanced pleiotropy.

In the main analyses, estimates were calculated using the radial inverse-variance weighted (IVW) approach with modified second-order weights in an iterative way [19]. If MR assumptions are met, this method provides the highest statistical power and accounts also for balanced pleiotropy. In each iteration step, pleiotropic SNPs were quantified and detected by calculating and testing the SNP-specific Cochran’s *Q*-statistics (based on a type I error *α*_*Q*_ = 0.01) as well as by graphical evaluation. Moreover, we assessed all identified heterogeneous SNPs responsible for possible unbalanced horizontal pleiotropy using PhenoScanner database [20, 21] (Additional file 1: Tables S5 to S8). To account for different patterns of pleiotropy, sensitivity analyses were applied to the final models that provided consistent point estimates under different assumptions. The weighted median approach allows up to 50% of the selected genetic instruments to be invalid. The weighted mode leads to consistent estimates, even if there are more than 50% invalid instruments. The MR-PRESSO method uses the global and SNP-specific observed residual sum of squares to test for general horizontal pleiotropy and outliers, respectively. Additionally, it provides a distortion test comparing estimates before and after outlier removal. As a final step, we applied a many weak instrument analysis using the MR-RAPS approach with a robust loss function and consideration of overdispersion.

Estimates were presented as odds ratios (ORs) with 95% confidence intervals (CIs) and can be interpreted as the average change in the outcome per 2.72-fold increase in the prevalence of the respective binary exposure. It is important to note that since the estimates have no clear interpretation except for the direction, they were only used to test whether a causal effect exists [22]. The type I error was set to *α* = 0.05. Due to multiple testing *P*-values were adjusted by the Benjamini-Hochberg procedure (Additional file 1: Table S9). All analyses were performed using the open-source statistical software R (version: 4.1.0).

All authors had access to the study data and reviewed and approved the final manuscript.

## Results

Estimates presented hereafter were obtained by the radial IVW approach with modified second-order weights as the principal analysis. Given *P*-values (*P*_*adj*_) were adjusted for multiple testing.

**Fig. 1:**
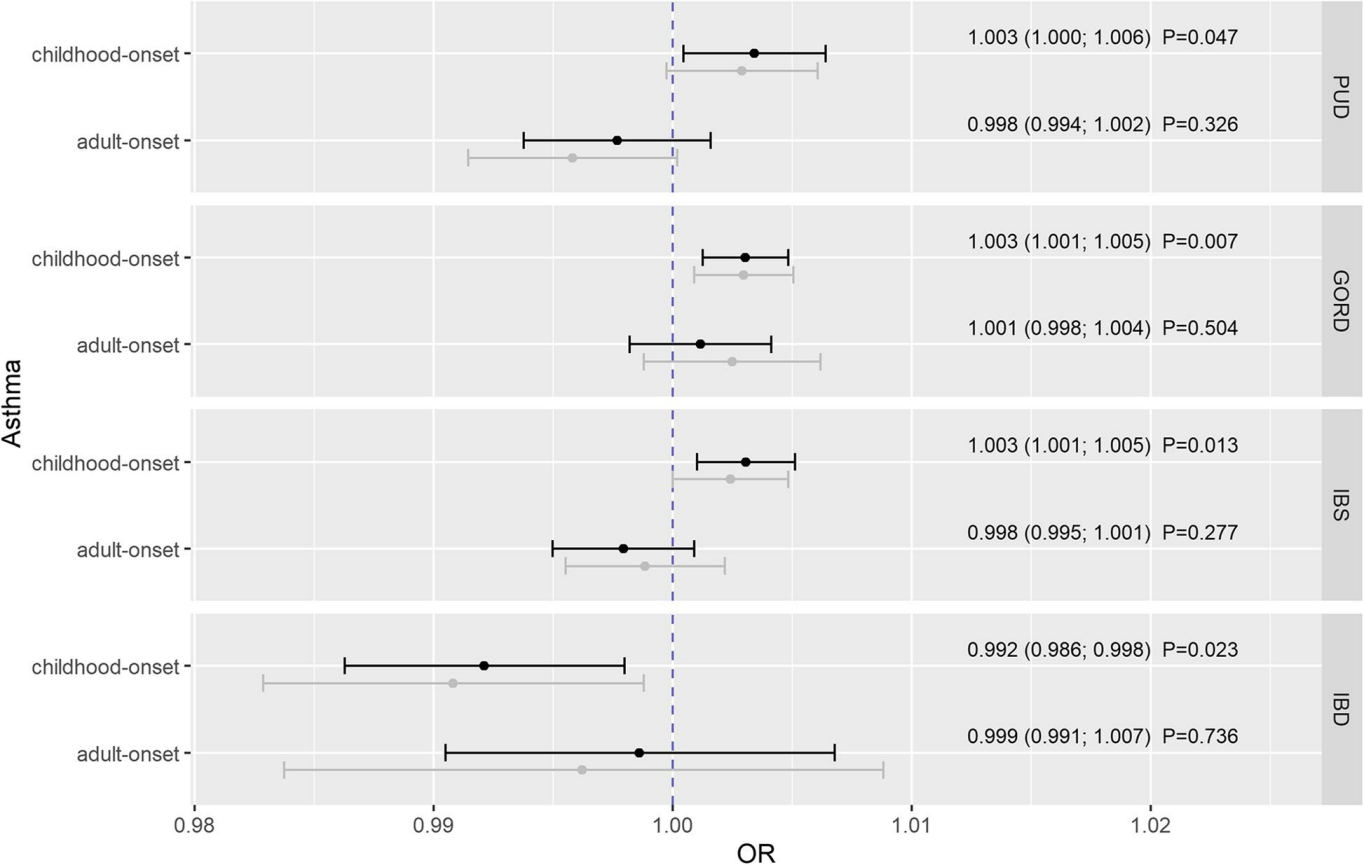
Estimates given as odds ratios (ORs) and 95% confidence intervals for the effect of childhood- and adult-onset asthma on peptic ulcer disease (PUD), gastroesophageal reflux disease (GORD), irritable bowel syndrome (IBS), and inflammatory bowel disease (IBD). Estimates were derived by the inverse-variance weighted method with modified second-order weights. Reported *P*-values were adjusted for multiple testing using the Benjamini-Hochberg procedure. Gray estimates represent the results before and black estimates the results after outlier-removal. PUD, peptic ulcer disease; GORD, gastroesophageal reflux disease; IBS, irritable bowel syndrome; IBD, inflammatory bowel disease

After removal of pleiotropic SNPs according to the Additional file 1: Tables S5 to S8, genetically predicted childhood-onset asthma was positively related to PUD (*OR*_*IVW*_ =1.003, 95% CI: 1.000; 1.006, *P*_*adj*_ =0.047), GORD (*OR*_*IVW*_ =1.003, 95% CI: 1.001; 1.005, *P*_*adj*_ =0.007), and IBS (*OR*_*IVW*_ =1.003, 95% CI: 1.001; 1.005, *P*_*adj*_ =0.013), but inversely to IBD (*OR*_*IVW*_ =0.992, 95% CI: 0.986; 0.998, *P*_*adj*_ =0.023) (Fig. 1). However, no notable associations could be observed between genetically predicted adult-onset asthma and the mentioned gastrointestinal disorders (Fig. 1).

**Fig. 2:**
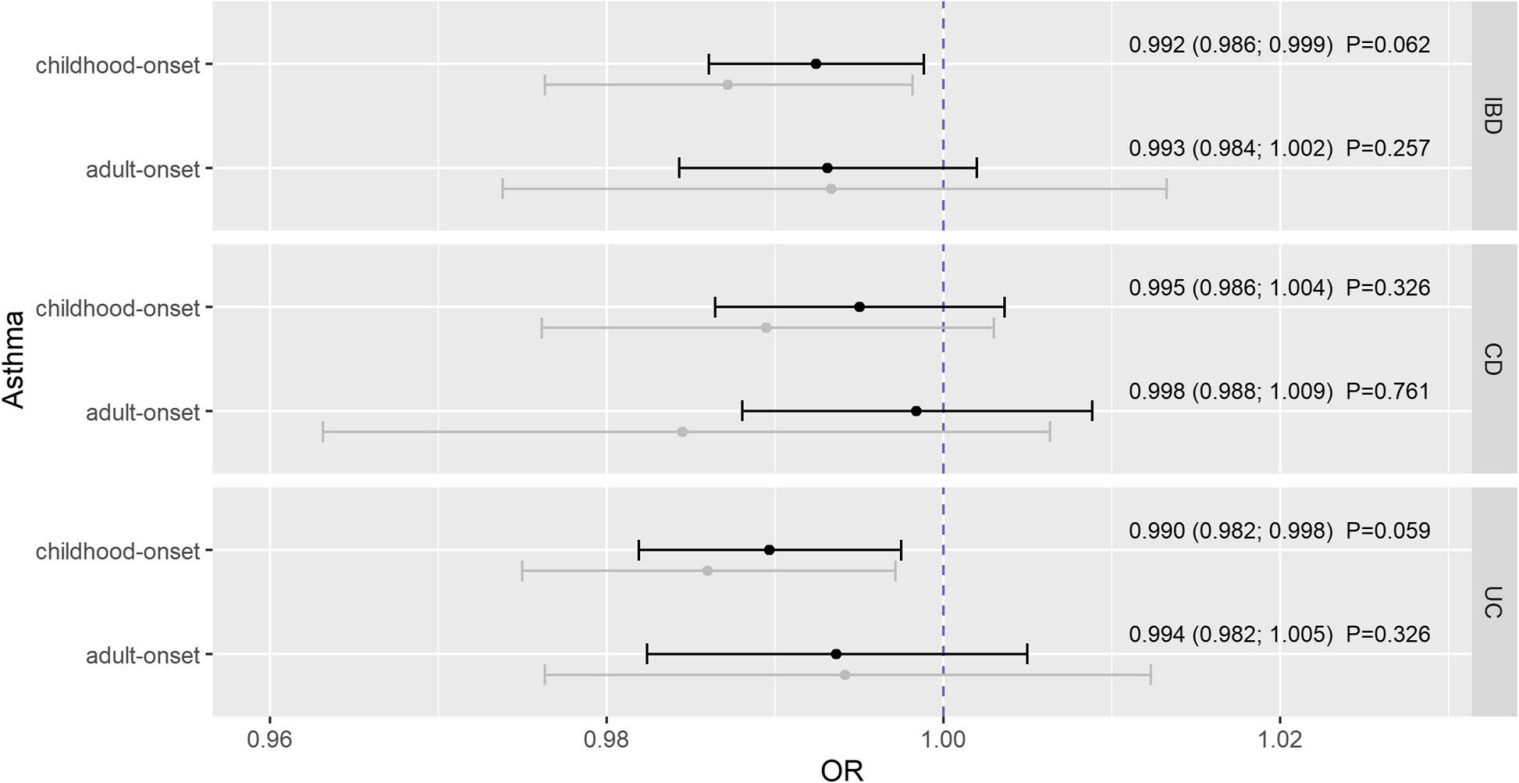
Estimates given as odds ratios (ORs) and 95% confidence intervals for the effect of childhood- and adult-onset asthma on peptic ulcer disease (PUD), gastroesophageal reflux disease (GORD), irritable bowel syndrome (IBS), and inflammatory bowel disease (IBD). Estimates were derived by the multiplicative random effects inverse-variance weighted method (except the effect of adult-onset asthma on ulcerative colitis that was estimated by the Wald atio approach). Reported *P*-values were adjusted for multiple testing using the Benjamini-Hochberg procedure. Gray estimates represent the results before and black estimates the results after outlier-removal. IBD, inflammatory bowel disease; CD, Crohn’s disease; UC, ulcerative colitis

The validation analysis confirmed our findings for the association between childhood-onset asthma and IBD showing similar and consistent effect estimates but with loss of statistical significance after correction for multiple testing (quantified by adjusted *P*-values in Fig. 2). Thus, genetically predicted childhood-onset asthma was inversely associated with IBD (*OR*_*IVW*_ =0.992, 95% CI: 0.986; 0.999, *P*_*adj*_ =0.062) (Fig. 2). The same applies to the relationship between childhood-onset asthma and UC (*OR*_*IVW*_ =0.990, 95% CI: 0.982; 0.998, *P*_*adj*_ =0.059), while no notable association could be observed with CD (*OR*_*IVW*_ =0.995, 95% CI: 0.986; 1.004, *P*_*adj*_ =0.326). Again, no associations could be observed between adult-onset asthma and IBD, CD, and UC (Fig. 2).

Except the association of genetically predicted childhood-onset asthma and UC, where the weighted mode led to an inconsistent point estimate, and despite of some heterogeneity quantified by the *Q*-statistics, the results were supported by the pleiotropic-robust approaches within sensitivity analyses (Additional file 1: Figs. S1-S4, Tables S10 and S11). Beyond that, removal of heterogeneous SNPs within the iterative radial approach did not substantially change the effect estimates (Figs. 1 and 2).

## Discussion

This is the first study showing evidence that childhood-onset asthma increases the risk for PUD, GORD, and IBS but decreases the risk for IBD (which may mainly be attributed to UC) later in life. The findings were supported by consistent magnitude and direction of robust methods. No conclusions were possible regarding the impact of adult-onset asthma on these gastrointestinal disorders due to weak effect estimates and inconsistencies within sensitivity analyses.

In observational studies, a frequent co-occurrence of asthma with IBD was reported [9, 12, 23]. A population-based Canadian case-control study using health administrative data including 3087 cases with Crohn’s disease, 2377 cases with ulcerative colitis, and 402,800 control subjects found that asthma was associated with Crohn’s disease (adjusted OR 1.45; 95% CI 1.31–1.60) and with early and late-onset ulcerative colitis. There was no association between asthma and ulcerative colitis among subjects aged 17 to 40 years [23]. A systematic review and meta-analysis including 18 matched-control and cohort studies found a positive association of asthma with Crohn’s disease and ulcerative colitis [12]. In that study, the risk of asthma was increased in patients with UC, but in patients with existing asthma, no increased risk of UC was observed. The authors assumed that geographic differences and differences in study design might be responsible for the high degree of heterogeneity between the included studies [12]. While in some countries asthma was associated with a higher risk of Crohn’s disease, in other countries, a negative association between these two diseases was found [12]. The present MR study showed an inverse association between childhood but not adulthood asthma and IBD and thus contributes to the evidence on this topic.

Asthma is also frequently associated with IBS, a disease that manifests with variable and fluctuating symptoms, such as abdominal pain, bloating, headache, and muscle pain [24]. In a retrospective analysis from Taiwan using National Health Insurance data, a bidirectional association between asthma and IBS was reported [25]. The relative risk of IBS was 1.57 [95% CI = 1.47–1.68] in the asthma cohort in comparison to subjects without asthma [25]. A meta-analysis including 8 case-control and 2 cross-sectional studies concluded that the risk of asthma is significantly higher in IBS patients and vice versa [10]. Patients with asthma first had twice the risk of having IBS and patients with IBS first had twice the risk of asthma [10]. Our study confirmed a causal effect of asthma on IBS; however, this only applies to asthma in childhood and the occurrence of IBS later in life. No causal relationship could be found for adulthood onset asthma and IBS.

A number of studies reported on the association of GORD and asthma [11, 26]. Although there seems to be a strong relationship between these two diseases, most prior studies were cross-sectional or case-control studies, thus, the direction of causality remained undetermined [11]. In a systematic review including 28 studies, the sample size weighted prevalence of gastroesophageal symptoms in patients with asthma was 59.2% (in controls 38.1%) and the average prevalence of asthma in subjects with GORD was 4.6% (in controls 3.9%) [11]. The authors concluded that there is a clear paucity of data on the causal direction of the association. The present MR study found a positive causal association between childhood asthma and GORD in adulthood, a result that strengthens the evidence of a causal relationship and thus contributes to clarifying the direction of the relationship.

There are no observational studies on the association between asthma and PUD. The present MR analysis suggested a causal effect of asthma onset in childhood on the development of peptic ulcer later in life. No causal effect of asthma onset in adults on peptic ulcer risk was found.

Prior studies reported that obesity and psychological traits such as depression or anxiety are associated with functional gastrointestinal disorders, e.g., irritable bowel syndrome and gastroesophageal reflux disease GERD [27–30]. Also, previous genome-wide association studies (GWASs) on GERD have found genetic overlaps with established risk factors such as obesity and depression [31, 32]. Thus, in our MR analysis, bias due to pleiotropic effects by other pathways than the investigated cannot be entirely excluded. However, to reduce pleiotropy, we identified pleiotropic genetic variants within an iterative approach and confirmed them using the PhenoScanner database.

In that way, we excluded SNPs strongly associated with possible confounding factors like obesity and medication use and compared the results before and after outlier removal. Furthermore, we performed a number of sensitivity analyses, which failed to find evidence for unbalanced horizontal pleiotropy in the final models. In addition, we used a stringent selection threshold (*P* < 5 · 10^−8^) to minimize weak instrument bias.

Nowadays, asthma is recognized as a complex syndrome rather than a single disease and the mechanisms determining childhood asthma might not necessarily lead to asthma in adulthood [8]. A possible explanation for the present findings could be that the asthma phenotype in childhood is more uniform compared to asthma in adulthood, in which various mechanisms might be involved in the development of the disease [8]. These differences might result in more precise data with higher explained variance by genetic risk factors (${h}_g^2=$ 25.6% in childhood-onset vs. ${h}_g^2=$ 10.6% in adult-onset asthma [15]). Furthermore, regarding the earlier time point of disease manifestation and therefore lower susceptibility to reverse causation [33], the cause-effect-chain for individuals with childhood-onset asthma is more sustainable than in case of a disease onset later in life.

Several underlying mechanisms may explain the link between asthma onset in childhood and the different intestinal diseases. It has been postulated that early life exposures are associated with a predisposition towards respiratory diseases and with changes in the intestinal microbiota [7]. For example, recurrent antibiotic treatment has detrimental effects on the diversity of the microbiota in childhood [34] and is significantly associated with a later onset of asthma [35, 36] and—as shown in murine studies—with a loss of protection against respiratory viruses [37]. Other studies found that cesarean birth is accompanied by an alteration of the gut microbiome in early infancy [38] and a predisposition towards the onset of asthma in childhood [39]. Whether these early-life factors also impact on the airway microbiota has not been elucidated yet. There is no question that there is a cross-talk between the intestinal and airway microbiota compartments [6]. However, so far, the mechanisms how both compartments interact are not fully elucidated.

Our study has several strengths. The MR concept is less susceptible to unobserved confounding and reverse causality that are issues of observational studies. Outlier-assessment and a wide range of sensitivity analyses that accounted for different patterns of pleiotropy increased the robustness of the results and thus strengthened the evidence of our findings.

The study has also notable limitations. One-sample MR is known to have less statistical power compared to the two-sample MR [40]. However, in large biobanks, such as UK Biobank, the results from one- and two-sample MRs are shown to be similar except for the MR-Egger method [41]. Moreover, we were able to validate the results for IBD and investigate its subgroups using a summary statistics from a second GWAS in a two-sample setting. Another limitation is that the magnitude of provided estimates cannot be directly compared to estimates from observational studies. Thus, we were only able to compare the direction of the estimates and strengthen evidence for a causal relationship. Finally, sex-specific and ethnic-stratified analyses were not possible due to the lack of suitable summary level data.

## Conclusions

In conclusion, we found that genetically predicted asthma onset in childhood is causally associated with a higher risk of GORD, PUD, and IBS and a lower risk of IBD in adults from the general population. Physicians should pay increased attention to whether gastrointestinal disease develops in patients who had manifest asthma in childhood. The pathophysiologic mechanisms underlying the cross-talk between the gut and the lung have to be explored in further investigations.

## Supplementary Information

**Additional file 1: Fig. S1** Sensitivity analyses of the effect of childhood-onset asthma on gastrointestinal disorders. **Fig. S2** Sensitivity analyses of the effect of adult-onset asthma on gastrointestinal disorders. **Fig. S3** Sensitivity analyses of the effect of childhood-onset asthma on IBD including subtypes. **Fig. S4** Sensitivity analyses of the effect of adult-onset asthma on IBD including subtypes. **Table S1**. SNPs used in the analyses of the effect of childhood-onset asthma on gastrointestinal disorders. **Table S2**. SNPs used in the analyses of the effect of adult-onset asthma on gastrointestinal disorders. **Table S3**. SNPs used in the analyses of the effect of childhood-onset asthma on IBD including subtypes. **Table S4**. SNPs used in the analyses of the effect of adult-onset asthma on IBD including subtypes. **Table S5**. Identified pleiotropic SNPs of the effect of childhood-onset asthma on gastrointestinal disorders. **Table S6**. Identified pleiotropic SNPs of the effect of adult-onset asthma on gastrointestinal disorders. **Table S7**. Identified pleiotropic SNPs of the effect of childhood-onset asthma on IBD including subtypes. **Table S8**. Identified pleiotropic SNPs of the effect of adult-onset asthma on IBD including subtypes. **Table S9**. Estimates, standard errors, raw *P*-values, and Benjamini-Hochberg-adjusted *P*-values from the Mendelian randomization analyses. **Table S10**. Heterogeneity statistics of the effect of asthma on gastrointestinal disorders. **Table S11**. Heterogeneity statistics of the effect of asthma on IBD including subtypes.

## Data Availability

The present study is based on summary-level data that have been made publically available. Summary data from genome-wide association studies for childhood-onset and adult-onset asthma are available at https://genepi.qimr.edu.au/staff/manuelF/gwas_results/main.html [42, 43]. Data for gastrointestinal disorders can be obtained from https://cnsgenomics.com/content/data [44–47] and https://www.ibdgenetics.org [48].

## Abbreviations

CD: Crohn’s disease
CI: Confidence interval
GORD: Gastroesophageal reflux disease
GWAS: Genome-wide association study
IBD: Inflammatory bowel disease
IBS: Irritable bowel syndrome
MR: Mendelian randomization
OR: Odds ratio
PUD: Peptic ulcer disease
SNP: Single nucleotide polymorphism
UC: Ulcerative colitis
