# Supplementary material for: Asthma and the risk of gastrointestinal disorders: a Mendelian randomization study
Source: BMC Med. 2022 Mar 16;20:82. doi: 10.1186/s12916-022-02283-7 (PMC8925069; doi:10.1186/s12916-022-02283-7)
Supplement: Supplementary file 1 — Additional file 1: Fig. S1 Sensitivity analyses of the effect of childhood-onset asthma on gastrointestinal disorders. Fig. S2 Sensitivity analyses of the effect of adult-onset asthma on gastrointestinal disorders. Fig. S3 Sensitivity analyses of the effect of childhood-onset asthma on IBD including subtypes. Fig. S4 Sensitivity analyses of the effect of adult-onset asthma on IBD including subtypes. Table S1. SNPs used in the analyses of the effect of childhood-onset asthma on gastrointestinal disorders. Table S2. SNPs used in the analyses of the effect of adult-onset asthma on gastrointestinal disorders. Table S3. SNPs used in the analyses of the effect of childhood-onset asthma on IBD including subtypes. Table S4. SNPs used in the analyses of the effect of adult-onset asthma on IBD including subtypes. Table S5. Identified pleiotropic SNPs of the effect of childhood-onset asthma on gastrointestinal disorders. Table S6. Identified pleiotropic SNPs of the effect of adult-onset asthma on gastrointestinal disorders. Table S7. Identified pleiotropic SNPs of the effect of childhood-onset asthma on IBD including subtypes. Table S8. Identified pleiotropic SNPs of the effect of adult-onset asthma on IBD including subtypes. Table S9. Estimates, standard errors, raw P-values, and Benjamini-Hochberg-adjusted P-values from the Mendelian randomization analyses. Table S10. Heterogeneity statistics of the effect of asthma on gastrointestinal disorders. Table S11. Heterogeneity statistics of the effect of asthma on IBD including subtypes. [file 12916_2022_2283_MOESM1_ESM.docx]

**Asthma and the risk of gastrointestinal disorders: A Mendelian randomization study**

**First author:** Dennis Freuer

Additional file


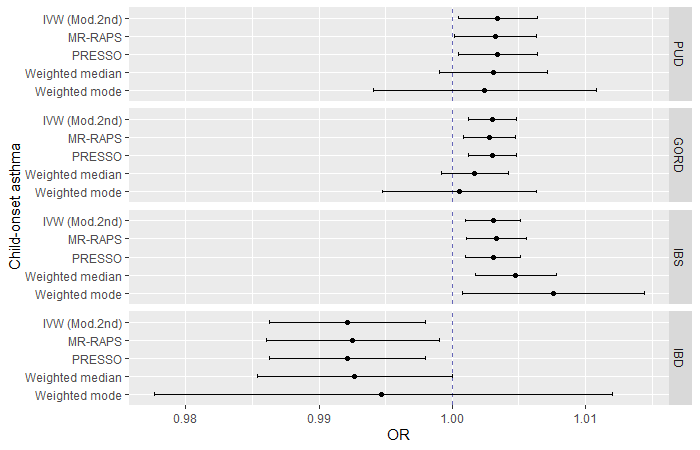


**Fig. S1** Mendelian Randomization sensitivity analyses of the effect of childhood-onset asthma on peptic ulcer disease (PUD), gastro-oesophageal reflux disease (GORD), irritable bowel syndrome (IBS) and inflammatory bowel disease (IBD). ORs can be interpreted as the average change in the outcome per 2.72-fold increase in the prevalence of the respective binary exposure.


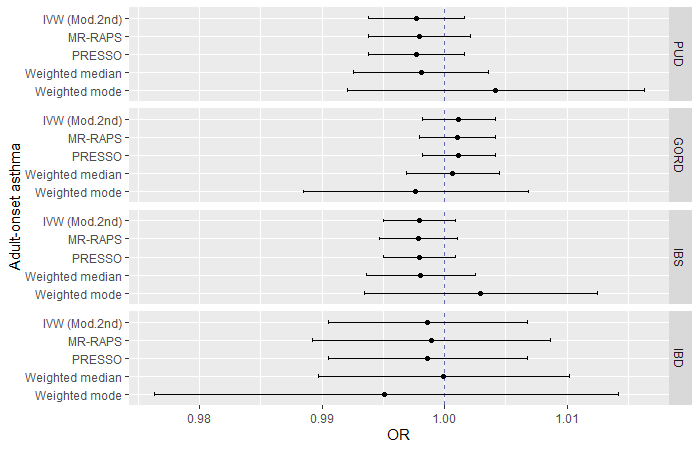


**Fig. S2** Mendelian Randomization sensitivity analyses of the effect of adult-onset asthma on peptic ulcer disease (PUD), gastro-oesophageal reflux disease (GORD), irritable bowel syndrome (IBS) and inflammatory bowel disease (IBD). ORs can be interpreted as the average change in the outcome per 2.72-fold increase in the prevalence of the respective binary exposure.


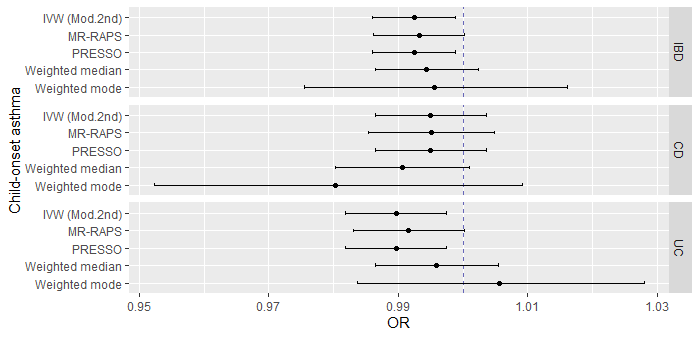


**Fig. S3** Mendelian Randomization sensitivity analyses of the effect of childhood-onset asthma on inflammatory bowel disease (IBD), Crohn´s disease (CD), and ulcerative colitis (UC). ORs can be interpreted as the average change in the outcome per 2.72-fold increase in the prevalence of the respective binary exposure.


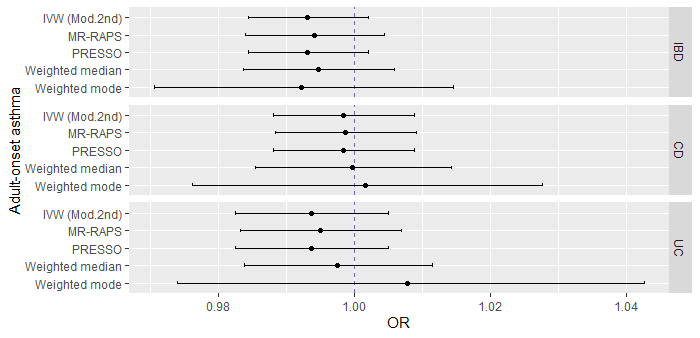


**Fig. S4** Mendelian Randomization sensitivity analyses of the effect of adult-onset asthma on inflammatory bowel disease (IBD), Crohn´s disease (CD), and ulcerative colitis (UC). ORs can be interpreted as the average change in the outcome per 2.72-fold increase in the prevalence of the respective binary exposure.

**Table S1** Genetic variants used in the analyses investigating a causal impact of genetically predicted childhood-onset asthma and gastrointestinal disorders

| **SNP** | **EA** | **OA** | **EAF** | $\boldsymbol{\beta}$**_expsr_** | **SE_expsr_** | $\boldsymbol{P}$**_expsr_** | $\boldsymbol{\beta}$**_otcm_** | **SE_otcm_** | $\boldsymbol{P}$**_otcm_** |
| --- | --- | --- | --- | --- | --- | --- | --- | --- | --- |
| **childhood-onset asthma on IBS** | | | | | | | | | |
| rs10118244 | C | T | 0.971414 | 1.21258 | 0.034716 | 2.8E-08 | 0.022883 | 0.026219 | 0.38 |
| rs10158467 | A | G | 0.714141 | 0.896019 | 0.012885 | 1.6E-17 | -0.00803 | 0.009663 | 0.41 |
| rs10187276 | T | C | 0.251175 | 1.11605 | 0.013347 | 1.9E-16 | 0.008374 | 0.009896 | 0.4 |
| rs10737105 | C | G | 0.202262 | 1.12268 | 0.01448 | 1.3E-15 | 0.003179 | 0.010803 | 0.77 |
| rs10786050 | A | G | 0.406743 | 0.917752 | 0.011754 | 2.8E-13 | 0.008085 | 0.008748 | 0.36 |
| rs10836538 | G | T | 0.651655 | 1.08321 | 0.012173 | 5.2E-11 | -0.00688 | 0.009066 | 0.45 |
| rs10995245 | G | A | 0.652823 | 0.931956 | 0.012168 | 7E-09 | 0.003899 | 0.009104 | 0.67 |
| rs11065979 | C | T | 0.558799 | 1.08502 | 0.011655 | 2.5E-12 | -0.00736 | 0.008697 | 0.4 |
| rs11071559 | C | T | 0.868457 | 1.18496 | 0.017078 | 2.9E-23 | 0.000455 | 0.012796 | 0.97 |
| rs11135015 | T | C | 0.670957 | 0.934697 | 0.012296 | 4E-08 | 0.004449 | 0.009176 | 0.63 |
| rs11236797 | C | A | 0.552697 | 0.823406 | 0.011634 | 1.3E-62 | 0.00295 | 0.008655 | 0.73 |
| rs11593589 | C | A | 0.457859 | 1.07221 | 0.011592 | 1.8E-09 | 0.002928 | 0.008628 | 0.73 |
| rs11658582 | C | G | 0.379145 | 0.912738 | 0.011907 | 1.7E-14 | 0.002825 | 0.008919 | 0.75 |
| rs117710327 | C | A | 0.931559 | 1.20548 | 0.023404 | 1.4E-15 | -0.00477 | 0.017985 | 0.79 |
| rs12023876 | G | T | 0.667519 | 1.07987 | 0.01226 | 3.7E-10 | 0.012363 | 0.009154 | 0.18 |
| rs12123821 | C | T | 0.953569 | 0.656525 | 0.027448 | 4.8E-53 | 0.005564 | 0.020259 | 0.78 |
| rs12365699 | G | A | 0.831274 | 1.14126 | 0.015499 | 1.5E-17 | 0.013202 | 0.011671 | 0.26 |
| rs12531500 | A | G | 0.565634 | 1.10309 | 0.011689 | 4.7E-17 | -0.02117 | 0.008719 | 0.015 |
| rs12657787 | G | A | 0.847423 | 0.849237 | 0.01615 | 4.6E-24 | 0.012008 | 0.012093 | 0.32 |
| rs12750027 | G | A | 0.933791 | 0.869787 | 0.023242 | 1.9E-09 | 0.016413 | 0.017344 | 0.34 |
| rs12935657 | G | A | 0.749187 | 1.15436 | 0.013371 | 6.9E-27 | 0.009903 | 0.010082 | 0.33 |
| rs12964116 | A | G | 0.963474 | 0.773634 | 0.030811 | 8.1E-17 | 0.010559 | 0.023185 | 0.65 |
| rs12965763 | G | A | 0.77782 | 0.909338 | 0.013953 | 9.7E-12 | -0.00078 | 0.010358 | 0.94 |
| rs13277355 | A | G | 0.274331 | 1.10297 | 0.012996 | 4.7E-14 | 0.015052 | 0.009656 | 0.12 |
| rs143326447 | T | C | 0.881359 | 0.902426 | 0.017883 | 9.4E-09 | 0.008789 | 0.013326 | 0.51 |
| rs150707349 | A | G | 0.96926 | 1.21721 | 0.034805 | 1.6E-08 | 0.020644 | 0.027231 | 0.45 |
| rs1612986 | T | C | 0.816653 | 0.856328 | 0.015037 | 6E-25 | -0.02318 | 0.011141 | 0.037 |
| rs1617333 | A | G | 0.60217 | 1.09812 | 0.011863 | 3E-15 | 0.008701 | 0.008879 | 0.33 |
| rs1655558 | T | G | 0.454064 | 0.919987 | 0.011706 | 1E-12 | -0.00713 | 0.008793 | 0.42 |
| rs16903574 | C | G | 0.924845 | 0.839851 | 0.022396 | 6.5E-15 | -0.01908 | 0.016789 | 0.26 |
| rs17103286 | A | G | 0.478517 | 0.926643 | 0.011603 | 5.2E-11 | -0.01605 | 0.008647 | 0.063 |
| rs17202120 | A | G | 0.982218 | 1.34477 | 0.043928 | 1.5E-11 | 0.082397 | 0.034447 | 0.017 |
| rs1806656 | C | G | 0.681098 | 1.08202 | 0.012509 | 2.9E-10 | 0.010003 | 0.009453 | 0.29 |
| rs1837253 | T | C | 0.262925 | 0.846361 | 0.013128 | 5.5E-37 | 6.63E-05 | 0.009807 | 0.99 |
| rs188074962 | G | A | 0.355239 | 1.11246 | 0.012173 | 2E-18 | -0.00484 | 0.009112 | 0.6 |
| rs1885013 | G | A | 0.281984 | 1.09787 | 0.012849 | 3.7E-13 | 0.003171 | 0.009551 | 0.74 |
| rs1887704 | C | G | 0.32215 | 0.907394 | 0.012388 | 4.3E-15 | -0.01018 | 0.009299 | 0.27 |
| rs1893380 | G | C | 0.6208 | 0.92337 | 0.011913 | 2.2E-11 | 0.000974 | 0.008863 | 0.91 |
| rs1962588 | T | G | 0.791595 | 0.922422 | 0.014396 | 2E-08 | 0.004425 | 0.010859 | 0.68 |
| rs2052690 | G | T | 0.712557 | 0.928894 | 0.012785 | 8E-09 | 0.009612 | 0.009566 | 0.31 |
| rs2066844 | C | T | 0.953476 | 0.853589 | 0.027433 | 7.9E-09 | 0.008814 | 0.020275 | 0.66 |
| rs2070901 | G | T | 0.731764 | 0.914937 | 0.013062 | 1E-11 | 0.014688 | 0.009737 | 0.13 |
| rs2202750 | T | C | 0.381027 | 1.11173 | 0.011896 | 5.4E-19 | -0.01285 | 0.008862 | 0.15 |
| rs2299012 | A | C | 0.808317 | 0.839763 | 0.014686 | 1.3E-32 | 0.001938 | 0.010929 | 0.86 |
| rs230495 | A | G | 0.412214 | 0.936226 | 0.011749 | 2E-08 | -0.01063 | 0.008789 | 0.23 |
| rs2738783 | T | G | 0.212924 | 0.919593 | 0.014227 | 3.8E-09 | -0.00184 | 0.010666 | 0.86 |
| rs274943 | T | C | 0.516893 | 1.07957 | 0.01159 | 4E-11 | 0.00859 | 0.008656 | 0.32 |
| rs2765414 | T | G | 0.225532 | 1.09099 | 0.013821 | 2.9E-10 | -0.02722 | 0.010349 | 0.0085 |
| rs2766667 | T | C | 0.256063 | 1.07946 | 0.013289 | 8.7E-09 | -0.0041 | 0.00989 | 0.68 |
| rs2918302 | G | A | 0.846221 | 0.903596 | 0.016049 | 2.7E-10 | 0.00992 | 0.011987 | 0.41 |
| rs2955118 | G | A | 0.725936 | 1.08175 | 0.013029 | 1.6E-09 | 0.022722 | 0.009784 | 0.02 |
| rs3097670 | G | C | 0.878406 | 1.15087 | 0.017894 | 4.1E-15 | 0.008899 | 0.013528 | 0.51 |
| rs3122929 | C | T | 0.600485 | 0.876303 | 0.011843 | 7.2E-29 | 0.005351 | 0.00886 | 0.55 |
| rs340931 | G | A | 0.327612 | 0.905216 | 0.012327 | 6.6E-16 | 0.012239 | 0.009173 | 0.18 |
| rs34290285 | G | A | 0.739377 | 1.19778 | 0.013168 | 9.5E-43 | 0.006627 | 0.009872 | 0.5 |
| rs35570272 | G | T | 0.604956 | 0.903966 | 0.011885 | 2E-17 | 0.006964 | 0.00889 | 0.43 |
| rs3785356 | C | T | 0.703372 | 0.896086 | 0.012727 | 6.6E-18 | -0.0038 | 0.009512 | 0.69 |
| rs3856439 | C | T | 0.656887 | 1.11341 | 0.012209 | 1.4E-18 | 0.000819 | 0.009156 | 0.93 |
| rs42403 | C | T | 0.887006 | 0.869917 | 0.018294 | 2.6E-14 | -0.01922 | 0.01354 | 0.16 |
| rs4319131 | A | G | 0.544753 | 1.07054 | 0.011651 | 4.9E-09 | 0.007581 | 0.008708 | 0.38 |
| rs45613035 | T | C | 0.903289 | 0.833266 | 0.019553 | 1.1E-20 | -0.01083 | 0.01441 | 0.45 |
| rs4574025 | C | T | 0.465373 | 0.915721 | 0.011658 | 4.3E-14 | 0.008721 | 0.008708 | 0.32 |
| rs4722758 | C | G | 0.800574 | 0.887831 | 0.014435 | 1.7E-16 | -0.00992 | 0.010709 | 0.35 |
| rs4792846 | G | A | 0.459444 | 1.08451 | 0.011645 | 3.2E-12 | 0.012644 | 0.008701 | 0.15 |
| rs4795399 | T | C | 0.530853 | 1.30283 | 0.011583 | 1.9E-115 | -0.00101 | 0.00862 | 0.91 |
| rs479844 | A | G | 0.446616 | 0.895698 | 0.011623 | 2.6E-21 | -0.00911 | 0.00866 | 0.29 |
| rs4916534 | A | G | 0.91899 | 1.12664 | 0.021354 | 2.3E-08 | 0.010276 | 0.016156 | 0.52 |
| rs4917131 | A | G | 0.190569 | 1.08637 | 0.014876 | 2.6E-08 | 0.006843 | 0.01117 | 0.54 |
| rs55743914 | C | T | 0.757827 | 0.904782 | 0.013575 | 1.7E-13 | 0.003415 | 0.010158 | 0.74 |
| rs56328339 | C | T | 0.836301 | 1.10129 | 0.015672 | 7.5E-10 | 0.011786 | 0.01176 | 0.32 |
| rs61584523 | G | C | 0.961081 | 1.18425 | 0.030532 | 3E-08 | 0.04279 | 0.023519 | 0.069 |
| rs61815559 | A | T | 0.966971 | 0.633937 | 0.033074 | 3.3E-43 | 0.00718 | 0.024878 | 0.77 |
| rs62408233 | G | A | 0.643957 | 1.11297 | 0.01205 | 6.5E-19 | -0.00306 | 0.008986 | 0.73 |
| rs6927172 | C | G | 0.779472 | 1.08976 | 0.013934 | 6.9E-10 | 0.008363 | 0.010423 | 0.42 |
| rs6954667 | C | A | 0.692969 | 0.931182 | 0.012569 | 1.4E-08 | 0.011532 | 0.009371 | 0.22 |
| rs705700 | T | C | 0.57995 | 0.90236 | 0.011723 | 1.9E-18 | 0.016631 | 0.008734 | 0.057 |
| rs71421264 | C | G | 0.415631 | 0.925104 | 0.011814 | 4.4E-11 | -0.00374 | 0.008869 | 0.67 |
| rs7209400 | C | T | 0.455041 | 0.924102 | 0.011658 | 1.3E-11 | 0.008022 | 0.008714 | 0.36 |
| rs72743461 | C | A | 0.767112 | 0.836304 | 0.013702 | 6.7E-39 | -0.00819 | 0.010106 | 0.42 |
| rs72823641 | T | A | 0.857876 | 1.34076 | 0.016524 | 1.8E-70 | 0.005778 | 0.01251 | 0.64 |
| rs73192661 | C | T | 0.549501 | 0.881349 | 0.011646 | 2.1E-27 | 0.005675 | 0.008693 | 0.51 |
| rs7582757 | A | G | 0.524664 | 0.937076 | 0.011573 | 2E-08 | 0.009865 | 0.008648 | 0.25 |
| rs7626218 | A | T | 0.602573 | 1.08332 | 0.011823 | 1.3E-11 | 0.006137 | 0.008828 | 0.49 |
| rs7848215 | C | T | 0.740551 | 0.796999 | 0.013274 | 1.7E-65 | -0.00646 | 0.009932 | 0.52 |
| rs7975763 | C | T | 0.793332 | 1.08807 | 0.014324 | 3.8E-09 | 0.047757 | 0.010853 | 0.000011 |
| rs835673 | T | C | 0.265721 | 0.931038 | 0.013093 | 4.8E-08 | 0.01139 | 0.009752 | 0.24 |
| rs892225 | A | G | 0.618623 | 0.925896 | 0.012027 | 1.5E-10 | -0.0229 | 0.009036 | 0.011 |
| rs912425 | G | A | 0.239061 | 1.08376 | 0.01359 | 3.2E-09 | -0.01447 | 0.010167 | 0.15 |
| rs9372120 | T | G | 0.7934 | 0.904475 | 0.014294 | 2.2E-12 | 0.009442 | 0.010647 | 0.38 |
| **childhood-onset asthma on IBD** | | | | | | | | | |
| rs10118244 | C | T | 0.971414 | 1.21258 | 0.034716 | 2.8E-08 | 0.051119 | 0.052458 | 0.33 |
| rs10158467 | A | G | 0.714141 | 0.896019 | 0.012885 | 1.6E-17 | -0.00069 | 0.019087 | 0.97 |
| rs10187276 | T | C | 0.251175 | 1.11605 | 0.013347 | 1.9E-16 | -0.06903 | 0.019891 | 0.00052 |
| rs10737105 | C | G | 0.202262 | 1.12268 | 0.01448 | 1.3E-15 | -0.02042 | 0.021458 | 0.34 |
| rs10786050 | A | G | 0.406743 | 0.917752 | 0.011754 | 2.8E-13 | -0.02599 | 0.017311 | 0.13 |
| rs10836538 | G | T | 0.651655 | 1.08321 | 0.012173 | 5.2E-11 | 0.044312 | 0.018022 | 0.014 |
| rs10995245 | G | A | 0.652823 | 0.931956 | 0.012168 | 7E-09 | 0.00909 | 0.017975 | 0.61 |
| rs11065979 | C | T | 0.558799 | 1.08502 | 0.011655 | 2.5E-12 | -0.0228 | 0.017144 | 0.18 |
| rs11071559 | C | T | 0.868457 | 1.18496 | 0.017078 | 2.9E-23 | 0.042109 | 0.02563 | 0.1 |
| rs11135015 | T | C | 0.670957 | 0.934697 | 0.012296 | 4E-08 | -0.05433 | 0.017932 | 0.0024 |
| rs11236797 | C | A | 0.552697 | 0.823406 | 0.011634 | 1.3E-62 | -0.10465 | 0.017004 | 7.5E-10 |
| rs11593589 | C | A | 0.457859 | 1.07221 | 0.011592 | 1.8E-09 | -0.03691 | 0.017049 | 0.03 |
| rs11658582 | C | G | 0.379145 | 0.912738 | 0.011907 | 1.7E-14 | -0.03323 | 0.017675 | 0.06 |
| rs117710327 | C | A | 0.931559 | 1.20548 | 0.023404 | 1.4E-15 | -0.05286 | 0.03476 | 0.13 |
| rs12023876 | G | T | 0.667519 | 1.07987 | 0.01226 | 3.7E-10 | -0.02397 | 0.017955 | 0.18 |
| rs12123821 | C | T | 0.953569 | 0.656525 | 0.027448 | 4.8E-53 | -0.03501 | 0.039267 | 0.37 |
| rs12365699 | G | A | 0.831274 | 1.14126 | 0.015499 | 1.5E-17 | 0.024089 | 0.023116 | 0.3 |
| rs12531500 | A | G | 0.565634 | 1.10309 | 0.011689 | 4.7E-17 | -0.01454 | 0.017205 | 0.4 |
| rs12657787 | G | A | 0.847423 | 0.849237 | 0.01615 | 4.6E-24 | -0.00625 | 0.02372 | 0.79 |
| rs12750027 | G | A | 0.933791 | 0.869787 | 0.023242 | 1.9E-09 | 0.00679 | 0.034094 | 0.84 |
| rs12935657 | G | A | 0.749187 | 1.15436 | 0.013371 | 6.9E-27 | 0.004477 | 0.01987 | 0.82 |
| rs12964116 | A | G | 0.963474 | 0.773634 | 0.030811 | 8.1E-17 | 0.027251 | 0.046109 | 0.55 |
| rs12965763 | G | A | 0.77782 | 0.909338 | 0.013953 | 9.7E-12 | -0.02479 | 0.020306 | 0.22 |
| rs13277355 | A | G | 0.274331 | 1.10297 | 0.012996 | 4.7E-14 | -0.03591 | 0.019257 | 0.062 |
| rs143326447 | T | C | 0.881359 | 0.902426 | 0.017883 | 9.4E-09 | 0.047804 | 0.026682 | 0.073 |
| rs150707349 | A | G | 0.96926 | 1.21721 | 0.034805 | 1.6E-08 | 0.084943 | 0.055384 | 0.13 |
| rs1612986 | T | C | 0.816653 | 0.856328 | 0.015037 | 6E-25 | -0.0202 | 0.021983 | 0.36 |
| rs1617333 | A | G | 0.60217 | 1.09812 | 0.011863 | 3E-15 | 0.00166 | 0.017503 | 0.92 |
| rs1655558 | T | G | 0.454064 | 0.919987 | 0.011706 | 1E-12 | 0.028557 | 0.017321 | 0.099 |
| rs16903574 | C | G | 0.924845 | 0.839851 | 0.022396 | 6.5E-15 | 0.055664 | 0.034135 | 0.1 |
| rs17103286 | A | G | 0.478517 | 0.926643 | 0.011603 | 5.2E-11 | 0.005524 | 0.01705 | 0.75 |
| rs17202120 | A | G | 0.982218 | 1.34477 | 0.043928 | 1.5E-11 | -0.09072 | 0.062878 | 0.15 |
| rs1806656 | C | G | 0.681098 | 1.08202 | 0.012509 | 2.9E-10 | 0.051826 | 0.018797 | 0.0058 |
| rs1837253 | T | C | 0.262925 | 0.846361 | 0.013128 | 5.5E-37 | -0.00518 | 0.019369 | 0.79 |
| rs188074962 | G | A | 0.355239 | 1.11246 | 0.012173 | 2E-18 | -0.02055 | 0.018017 | 0.25 |
| rs1885013 | G | A | 0.281984 | 1.09787 | 0.012849 | 3.7E-13 | -0.02024 | 0.018935 | 0.29 |
| rs1887704 | C | G | 0.32215 | 0.907394 | 0.012388 | 4.3E-15 | 0.022093 | 0.018246 | 0.23 |
| rs1893380 | G | C | 0.6208 | 0.92337 | 0.011913 | 2.2E-11 | -0.02056 | 0.017443 | 0.24 |
| rs1962588 | T | G | 0.791595 | 0.922422 | 0.014396 | 2E-08 | 0.012958 | 0.021478 | 0.55 |
| rs2052690 | G | T | 0.712557 | 0.928894 | 0.012785 | 8E-09 | -0.03357 | 0.018708 | 0.073 |
| rs2066844 | C | T | 0.953476 | 0.853589 | 0.027433 | 7.9E-09 | -0.12346 | 0.03774 | 0.0011 |
| rs2070901 | G | T | 0.731764 | 0.914937 | 0.013062 | 1E-11 | 0.003479 | 0.019165 | 0.86 |
| rs2202750 | T | C | 0.381027 | 1.11173 | 0.011896 | 5.4E-19 | -0.05227 | 0.017568 | 0.0029 |
| rs2299012 | A | C | 0.808317 | 0.839763 | 0.014686 | 1.3E-32 | -0.00012 | 0.021546 | 1 |
| rs230495 | A | G | 0.412214 | 0.936226 | 0.011749 | 2E-08 | 0.052755 | 0.01725 | 0.0022 |
| rs2738783 | T | G | 0.212924 | 0.919593 | 0.014227 | 3.8E-09 | -0.0911 | 0.021587 | 0.000024 |
| rs274943 | T | C | 0.516893 | 1.07957 | 0.01159 | 4E-11 | -0.00794 | 0.017074 | 0.64 |
| rs2765414 | T | G | 0.225532 | 1.09099 | 0.013821 | 2.9E-10 | 0.010486 | 0.02023 | 0.6 |
| rs2766667 | T | C | 0.256063 | 1.07946 | 0.013289 | 8.7E-09 | 0.019609 | 0.019405 | 0.31 |
| rs2918302 | G | A | 0.846221 | 0.903596 | 0.016049 | 2.7E-10 | -0.0328 | 0.023319 | 0.16 |
| rs2955118 | G | A | 0.725936 | 1.08175 | 0.013029 | 1.6E-09 | -0.03756 | 0.01906 | 0.049 |
| rs3097670 | G | C | 0.878406 | 1.15087 | 0.017894 | 4.1E-15 | 0.055811 | 0.027162 | 0.04 |
| rs3122929 | C | T | 0.600485 | 0.876303 | 0.011843 | 7.2E-29 | -0.02744 | 0.017426 | 0.12 |
| rs340931 | G | A | 0.327612 | 0.905216 | 0.012327 | 6.6E-16 | -0.004 | 0.018144 | 0.83 |
| rs34290285 | G | A | 0.739377 | 1.19778 | 0.013168 | 9.5E-43 | -0.04317 | 0.019255 | 0.025 |
| rs35570272 | G | T | 0.604956 | 0.903966 | 0.011885 | 2E-17 | 0.047233 | 0.017618 | 0.0073 |
| rs3785356 | C | T | 0.703372 | 0.896086 | 0.012727 | 6.6E-18 | -0.05296 | 0.018586 | 0.0044 |
| rs3856439 | C | T | 0.656887 | 1.11341 | 0.012209 | 1.4E-18 | -0.01023 | 0.018034 | 0.57 |
| rs42403 | C | T | 0.887006 | 0.869917 | 0.018294 | 2.6E-14 | -0.00345 | 0.026848 | 0.9 |
| rs4319131 | A | G | 0.544753 | 1.07054 | 0.011651 | 4.9E-09 | 0.038808 | 0.017205 | 0.024 |
| rs45613035 | T | C | 0.903289 | 0.833266 | 0.019553 | 1.1E-20 | -0.05567 | 0.027928 | 0.046 |
| rs4574025 | C | T | 0.465373 | 0.915721 | 0.011658 | 4.3E-14 | -0.01199 | 0.017191 | 0.49 |
| rs4722758 | C | G | 0.800574 | 0.887831 | 0.014435 | 1.7E-16 | 0.045302 | 0.021463 | 0.035 |
| rs4792846 | G | A | 0.459444 | 1.08451 | 0.011645 | 3.2E-12 | 0.01506 | 0.017163 | 0.38 |
| rs4795399 | T | C | 0.530853 | 1.30283 | 0.011583 | 1.9E-115 | -0.05973 | 0.016979 | 0.00043 |
| rs479844 | A | G | 0.446616 | 0.895698 | 0.011623 | 2.6E-21 | -0.00916 | 0.017087 | 0.59 |
| rs4916534 | A | G | 0.91899 | 1.12664 | 0.021354 | 2.3E-08 | -0.00165 | 0.031735 | 0.96 |
| rs4917131 | A | G | 0.190569 | 1.08637 | 0.014876 | 2.6E-08 | -0.07392 | 0.022583 | 0.0011 |
| rs55743914 | C | T | 0.757827 | 0.904782 | 0.013575 | 1.7E-13 | -0.01902 | 0.019928 | 0.34 |
| rs56328339 | C | T | 0.836301 | 1.10129 | 0.015672 | 7.5E-10 | 0.052258 | 0.023524 | 0.026 |
| rs61584523 | G | C | 0.961081 | 1.18425 | 0.030532 | 3E-08 | 0.00148 | 0.04563 | 0.97 |
| rs61815559 | A | T | 0.966971 | 0.633937 | 0.033074 | 3.3E-43 | -0.02604 | 0.048343 | 0.59 |
| rs62408233 | G | A | 0.643957 | 1.11297 | 0.01205 | 6.5E-19 | 0.043432 | 0.017845 | 0.015 |
| rs6927172 | C | G | 0.779472 | 1.08976 | 0.013934 | 6.9E-10 | -0.11835 | 0.01988 | 2.6E-09 |
| rs6954667 | C | A | 0.692969 | 0.931182 | 0.012569 | 1.4E-08 | -0.00633 | 0.018429 | 0.73 |
| rs705700 | T | C | 0.57995 | 0.90236 | 0.011723 | 1.9E-18 | 0.015858 | 0.017232 | 0.36 |
| rs71421264 | C | G | 0.415631 | 0.925104 | 0.011814 | 4.4E-11 | 0.004068 | 0.017487 | 0.82 |
| rs7209400 | C | T | 0.455041 | 0.924102 | 0.011658 | 1.3E-11 | -0.04848 | 0.017237 | 0.0049 |
| rs72743461 | C | A | 0.767112 | 0.836304 | 0.013702 | 6.7E-39 | -0.0848 | 0.019552 | 0.000014 |
| rs72823641 | T | A | 0.857876 | 1.34076 | 0.016524 | 1.8E-70 | 0.00155 | 0.02465 | 0.95 |
| rs73192661 | C | T | 0.549501 | 0.881349 | 0.011646 | 2.1E-27 | 0.029436 | 0.017174 | 0.087 |
| rs7582757 | A | G | 0.524664 | 0.937076 | 0.011573 | 2E-08 | -0.03226 | 0.017046 | 0.058 |
| rs7626218 | A | T | 0.602573 | 1.08332 | 0.011823 | 1.3E-11 | -0.00461 | 0.017401 | 0.79 |
| rs7848215 | C | T | 0.740551 | 0.796999 | 0.013274 | 1.7E-65 | -0.01827 | 0.019538 | 0.35 |
| rs7975763 | C | T | 0.793332 | 1.08807 | 0.014324 | 3.8E-09 | -0.00538 | 0.021113 | 0.8 |
| rs835673 | T | C | 0.265721 | 0.931038 | 0.013093 | 4.8E-08 | 0.06318 | 0.019008 | 0.00089 |
| rs892225 | A | G | 0.618623 | 0.925896 | 0.012027 | 1.5E-10 | 0.008776 | 0.017887 | 0.62 |
| rs912425 | G | A | 0.239061 | 1.08376 | 0.01359 | 3.2E-09 | 0.007862 | 0.019953 | 0.69 |
| rs9372120 | T | G | 0.7934 | 0.904475 | 0.014294 | 2.2E-12 | 0.040517 | 0.021194 | 0.056 |
| **childhood-onset asthma on PUD** | | | | | | | | | |
| rs10118244 | C | T | 0.971414 | 1.21258 | 0.034716 | 2.8E-08 | 0.063932 | 0.034494 | 0.064 |
| rs10158467 | A | G | 0.714141 | 0.896019 | 0.012885 | 1.6E-17 | -0.01367 | 0.012463 | 0.27 |
| rs10187276 | T | C | 0.251175 | 1.11605 | 0.013347 | 1.9E-16 | 0.035371 | 0.0127 | 0.0054 |
| rs10737105 | C | G | 0.202262 | 1.12268 | 0.01448 | 1.3E-15 | 0.00501 | 0.013943 | 0.72 |
| rs10786050 | A | G | 0.406743 | 0.917752 | 0.011754 | 2.8E-13 | -0.00556 | 0.011309 | 0.62 |
| rs10836538 | G | T | 0.651655 | 1.08321 | 0.012173 | 5.2E-11 | 0.005776 | 0.011728 | 0.62 |
| rs10995245 | G | A | 0.652823 | 0.931956 | 0.012168 | 7E-09 | 0.007757 | 0.011763 | 0.51 |
| rs11065979 | C | T | 0.558799 | 1.08502 | 0.011655 | 2.5E-12 | 0.004697 | 0.011239 | 0.68 |
| rs11071559 | C | T | 0.868457 | 1.18496 | 0.017078 | 2.9E-23 | 0.030167 | 0.016696 | 0.071 |
| rs11135015 | T | C | 0.670957 | 0.934697 | 0.012296 | 4E-08 | 0.015966 | 0.011872 | 0.18 |
| rs11236797 | C | A | 0.552697 | 0.823406 | 0.011634 | 1.3E-62 | -0.0085 | 0.011171 | 0.45 |
| rs11593589 | C | A | 0.457859 | 1.07221 | 0.011592 | 1.8E-09 | 0.003327 | 0.011141 | 0.77 |
| rs11658582 | C | G | 0.379145 | 0.912738 | 0.011907 | 1.7E-14 | 0.01168 | 0.011506 | 0.31 |
| rs117710327 | C | A | 0.931559 | 1.20548 | 0.023404 | 1.4E-15 | -0.05195 | 0.022783 | 0.023 |
| rs12023876 | G | T | 0.667519 | 1.07987 | 0.01226 | 3.7E-10 | 0.025901 | 0.011848 | 0.029 |
| rs12123821 | C | T | 0.953569 | 0.656525 | 0.027448 | 4.8E-53 | 0.003344 | 0.026141 | 0.9 |
| rs12365699 | G | A | 0.831274 | 1.14126 | 0.015499 | 1.5E-17 | 0.045789 | 0.015229 | 0.0026 |
| rs12531500 | A | G | 0.565634 | 1.10309 | 0.011689 | 4.7E-17 | 0.000893 | 0.011273 | 0.94 |
| rs12657787 | G | A | 0.847423 | 0.849237 | 0.01615 | 4.6E-24 | 0.009113 | 0.015605 | 0.56 |
| rs12750027 | G | A | 0.933791 | 0.869787 | 0.023242 | 1.9E-09 | 0.001368 | 0.022268 | 0.95 |
| rs12935657 | G | A | 0.749187 | 1.15436 | 0.013371 | 6.9E-27 | -0.00384 | 0.012979 | 0.77 |
| rs12964116 | A | G | 0.963474 | 0.773634 | 0.030811 | 8.1E-17 | -0.01439 | 0.029629 | 0.63 |
| rs12965763 | G | A | 0.77782 | 0.909338 | 0.013953 | 9.7E-12 | -0.00443 | 0.013364 | 0.74 |
| rs13277355 | A | G | 0.274331 | 1.10297 | 0.012996 | 4.7E-14 | 0.00418 | 0.012495 | 0.74 |
| rs143326447 | T | C | 0.881359 | 0.902426 | 0.017883 | 9.4E-09 | 0.023236 | 0.017301 | 0.18 |
| rs150707349 | A | G | 0.96926 | 1.21721 | 0.034805 | 1.6E-08 | 0.059311 | 0.03579 | 0.097 |
| rs1612986 | T | C | 0.816653 | 0.856328 | 0.015037 | 6E-25 | -0.01718 | 0.014405 | 0.23 |
| rs1617333 | A | G | 0.60217 | 1.09812 | 0.011863 | 3E-15 | -0.02629 | 0.011427 | 0.021 |
| rs1655558 | T | G | 0.454064 | 0.919987 | 0.011706 | 1E-12 | -0.00252 | 0.011353 | 0.82 |
| rs16903574 | C | G | 0.924845 | 0.839851 | 0.022396 | 6.5E-15 | -0.03576 | 0.021526 | 0.097 |
| rs17103286 | A | G | 0.478517 | 0.926643 | 0.011603 | 5.2E-11 | 0.003828 | 0.011161 | 0.73 |
| rs17202120 | A | G | 0.982218 | 1.34477 | 0.043928 | 1.5E-11 | 0.000859 | 0.04297 | 0.98 |
| rs1806656 | C | G | 0.681098 | 1.08202 | 0.012509 | 2.9E-10 | -0.00826 | 0.01217 | 0.5 |
| rs1837253 | T | C | 0.262925 | 0.846361 | 0.013128 | 5.5E-37 | 0.013535 | 0.012626 | 0.28 |
| rs188074962 | G | A | 0.355239 | 1.11246 | 0.012173 | 2E-18 | -0.01506 | 0.011783 | 0.2 |
| rs1885013 | G | A | 0.281984 | 1.09787 | 0.012849 | 3.7E-13 | 0.000636 | 0.012339 | 0.96 |
| rs1887704 | C | G | 0.32215 | 0.907394 | 0.012388 | 4.3E-15 | -0.00492 | 0.011999 | 0.68 |
| rs1893380 | G | C | 0.6208 | 0.92337 | 0.011913 | 2.2E-11 | 0.003588 | 0.011449 | 0.75 |
| rs1962588 | T | G | 0.791595 | 0.922422 | 0.014396 | 2E-08 | 0.033052 | 0.014135 | 0.019 |
| rs2052690 | G | T | 0.712557 | 0.928894 | 0.012785 | 8E-09 | 0.006174 | 0.012345 | 0.62 |
| rs2066844 | C | T | 0.953476 | 0.853589 | 0.027433 | 7.9E-09 | -0.0065 | 0.026011 | 0.8 |
| rs2070901 | G | T | 0.731764 | 0.914937 | 0.013062 | 1E-11 | 0.023272 | 0.012599 | 0.065 |
| rs2202750 | T | C | 0.381027 | 1.11173 | 0.011896 | 5.4E-19 | 0.015744 | 0.011409 | 0.17 |
| rs2299012 | A | C | 0.808317 | 0.839763 | 0.014686 | 1.3E-32 | -0.0294 | 0.013987 | 0.036 |
| rs230495 | A | G | 0.412214 | 0.936226 | 0.011749 | 2E-08 | 0.004196 | 0.011336 | 0.71 |
| rs2738783 | T | G | 0.212924 | 0.919593 | 0.014227 | 3.8E-09 | -0.00407 | 0.013782 | 0.77 |
| rs274943 | T | C | 0.516893 | 1.07957 | 0.01159 | 4E-11 | 0.002679 | 0.011178 | 0.81 |
| rs2765414 | T | G | 0.225532 | 1.09099 | 0.013821 | 2.9E-10 | -0.03305 | 0.01339 | 0.014 |
| rs2766667 | T | C | 0.256063 | 1.07946 | 0.013289 | 8.7E-09 | -0.01487 | 0.012803 | 0.25 |
| rs2918302 | G | A | 0.846221 | 0.903596 | 0.016049 | 2.7E-10 | 0.021296 | 0.015538 | 0.17 |
| rs2955118 | G | A | 0.725936 | 1.08175 | 0.013029 | 1.6E-09 | 0.010226 | 0.012605 | 0.42 |
| rs3097670 | G | C | 0.878406 | 1.15087 | 0.017894 | 4.1E-15 | -0.00351 | 0.017394 | 0.84 |
| rs3122929 | C | T | 0.600485 | 0.876303 | 0.011843 | 7.2E-29 | 0.013991 | 0.01145 | 0.22 |
| rs340931 | G | A | 0.327612 | 0.905216 | 0.012327 | 6.6E-16 | 0.03048 | 0.01181 | 0.0099 |
| rs34290285 | G | A | 0.739377 | 1.19778 | 0.013168 | 9.5E-43 | 0.010916 | 0.012763 | 0.39 |
| rs35570272 | G | T | 0.604956 | 0.903966 | 0.011885 | 2E-17 | -0.00981 | 0.011463 | 0.39 |
| rs3785356 | C | T | 0.703372 | 0.896086 | 0.012727 | 6.6E-18 | -0.01354 | 0.01226 | 0.27 |
| rs3856439 | C | T | 0.656887 | 1.11341 | 0.012209 | 1.4E-18 | -0.01089 | 0.011804 | 0.36 |
| rs42403 | C | T | 0.887006 | 0.869917 | 0.018294 | 2.6E-14 | -0.0088 | 0.017542 | 0.62 |
| rs4319131 | A | G | 0.544753 | 1.07054 | 0.011651 | 4.9E-09 | 0.001723 | 0.011243 | 0.88 |
| rs45613035 | T | C | 0.903289 | 0.833266 | 0.019553 | 1.1E-20 | 0.003316 | 0.018703 | 0.86 |
| rs4574025 | C | T | 0.465373 | 0.915721 | 0.011658 | 4.3E-14 | 0.018752 | 0.011241 | 0.095 |
| rs4722758 | C | G | 0.800574 | 0.887831 | 0.014435 | 1.7E-16 | 0.00078 | 0.013867 | 0.96 |
| rs4792846 | G | A | 0.459444 | 1.08451 | 0.011645 | 3.2E-12 | 0.004368 | 0.011239 | 0.7 |
| rs4795399 | T | C | 0.530853 | 1.30283 | 0.011583 | 1.9E-115 | 0.002818 | 0.011132 | 0.8 |
| rs479844 | A | G | 0.446616 | 0.895698 | 0.011623 | 2.6E-21 | -0.00776 | 0.011183 | 0.49 |
| rs4916534 | A | G | 0.91899 | 1.12664 | 0.021354 | 2.3E-08 | -0.029 | 0.020551 | 0.16 |
| rs4917131 | A | G | 0.190569 | 1.08637 | 0.014876 | 2.6E-08 | 0.00137 | 0.014446 | 0.92 |
| rs55743914 | C | T | 0.757827 | 0.904782 | 0.013575 | 1.7E-13 | -0.00025 | 0.013106 | 0.98 |
| rs56328339 | C | T | 0.836301 | 1.10129 | 0.015672 | 7.5E-10 | 0.014572 | 0.015203 | 0.34 |
| rs61584523 | G | C | 0.961081 | 1.18425 | 0.030532 | 3E-08 | -0.01407 | 0.029667 | 0.64 |
| rs61815559 | A | T | 0.966971 | 0.633937 | 0.033074 | 3.3E-43 | -0.03585 | 0.031529 | 0.26 |
| rs62408233 | G | A | 0.643957 | 1.11297 | 0.01205 | 6.5E-19 | 0.006696 | 0.011618 | 0.56 |
| rs6927172 | C | G | 0.779472 | 1.08976 | 0.013934 | 6.9E-10 | 0.01343 | 0.013477 | 0.32 |
| rs6954667 | C | A | 0.692969 | 0.931182 | 0.012569 | 1.4E-08 | 0.015109 | 0.012109 | 0.21 |
| rs705700 | T | C | 0.57995 | 0.90236 | 0.011723 | 1.9E-18 | 0.016044 | 0.011279 | 0.15 |
| rs71421264 | C | G | 0.415631 | 0.925104 | 0.011814 | 4.4E-11 | -0.0027 | 0.011453 | 0.81 |
| rs7209400 | C | T | 0.455041 | 0.924102 | 0.011658 | 1.3E-11 | -0.01967 | 0.011265 | 0.081 |
| rs72743461 | C | A | 0.767112 | 0.836304 | 0.013702 | 6.7E-39 | 0.011965 | 0.013111 | 0.36 |
| rs72823641 | T | A | 0.857876 | 1.34076 | 0.016524 | 1.8E-70 | 0.018165 | 0.016225 | 0.26 |
| rs73192661 | C | T | 0.549501 | 0.881349 | 0.011646 | 2.1E-27 | -0.00135 | 0.011223 | 0.9 |
| rs7582757 | A | G | 0.524664 | 0.937076 | 0.011573 | 2E-08 | -0.00161 | 0.011165 | 0.89 |
| rs7626218 | A | T | 0.602573 | 1.08332 | 0.011823 | 1.3E-11 | -0.00464 | 0.011389 | 0.68 |
| rs7848215 | C | T | 0.740551 | 0.796999 | 0.013274 | 1.7E-65 | 0.017605 | 0.012894 | 0.17 |
| rs7975763 | C | T | 0.793332 | 1.08807 | 0.014324 | 3.8E-09 | 0.021263 | 0.013922 | 0.13 |
| rs835673 | T | C | 0.265721 | 0.931038 | 0.013093 | 4.8E-08 | 0.000965 | 0.012619 | 0.94 |
| rs892225 | A | G | 0.618623 | 0.925896 | 0.012027 | 1.5E-10 | -0.01098 | 0.011681 | 0.35 |
| rs912425 | G | A | 0.239061 | 1.08376 | 0.01359 | 3.2E-09 | 0.009735 | 0.013055 | 0.46 |
| rs9372120 | T | G | 0.7934 | 0.904475 | 0.014294 | 2.2E-12 | -0.01331 | 0.013665 | 0.33 |
| **childhood-onset asthma on GORD** | | | | | | | | | |
| rs10118244 | C | T | 0.971414 | 1.21258 | 0.034716 | 2.8E-08 | 0.020877 | 0.01946 | 0.28 |
| rs10158467 | A | G | 0.714141 | 0.896019 | 0.012885 | 1.6E-17 | -0.00044 | 0.007194 | 0.95 |
| rs10187276 | T | C | 0.251175 | 1.11605 | 0.013347 | 1.9E-16 | 0.013097 | 0.007351 | 0.075 |
| rs10737105 | C | G | 0.202262 | 1.12268 | 0.01448 | 1.3E-15 | 0.020068 | 0.008002 | 0.012 |
| rs10786050 | A | G | 0.406743 | 0.917752 | 0.011754 | 2.8E-13 | -0.00298 | 0.006509 | 0.65 |
| rs10836538 | G | T | 0.651655 | 1.08321 | 0.012173 | 5.2E-11 | 0.000795 | 0.006747 | 0.91 |
| rs10995245 | G | A | 0.652823 | 0.931956 | 0.012168 | 7E-09 | 0.001954 | 0.006766 | 0.77 |
| rs11065979 | C | T | 0.558799 | 1.08502 | 0.011655 | 2.5E-12 | -0.01568 | 0.006464 | 0.015 |
| rs11071559 | C | T | 0.868457 | 1.18496 | 0.017078 | 2.9E-23 | -0.00853 | 0.009492 | 0.37 |
| rs11135015 | T | C | 0.670957 | 0.934697 | 0.012296 | 4E-08 | 0.01099 | 0.006826 | 0.11 |
| rs11236797 | C | A | 0.552697 | 0.823406 | 0.011634 | 1.3E-62 | 0.002103 | 0.006434 | 0.74 |
| rs11593589 | C | A | 0.457859 | 1.07221 | 0.011592 | 1.8E-09 | 0.007328 | 0.006414 | 0.25 |
| rs11658582 | C | G | 0.379145 | 0.912738 | 0.011907 | 1.7E-14 | 0.011195 | 0.006627 | 0.091 |
| rs117710327 | C | A | 0.931559 | 1.20548 | 0.023404 | 1.4E-15 | 0.011031 | 0.013447 | 0.41 |
| rs12023876 | G | T | 0.667519 | 1.07987 | 0.01226 | 3.7E-10 | 0.007018 | 0.006799 | 0.3 |
| rs12123821 | C | T | 0.953569 | 0.656525 | 0.027448 | 4.8E-53 | 0.04312 | 0.015252 | 0.0047 |
| rs12365699 | G | A | 0.831274 | 1.14126 | 0.015499 | 1.5E-17 | 0.019075 | 0.008686 | 0.028 |
| rs12531500 | A | G | 0.565634 | 1.10309 | 0.011689 | 4.7E-17 | -0.00898 | 0.006486 | 0.17 |
| rs12657787 | G | A | 0.847423 | 0.849237 | 0.01615 | 4.6E-24 | -0.00677 | 0.008941 | 0.45 |
| rs12750027 | G | A | 0.933791 | 0.869787 | 0.023242 | 1.9E-09 | 0.017774 | 0.012887 | 0.17 |
| rs12935657 | G | A | 0.749187 | 1.15436 | 0.013371 | 6.9E-27 | -0.00181 | 0.007478 | 0.81 |
| rs12964116 | A | G | 0.963474 | 0.773634 | 0.030811 | 8.1E-17 | -0.00908 | 0.017112 | 0.6 |
| rs12965763 | G | A | 0.77782 | 0.909338 | 0.013953 | 9.7E-12 | -0.00397 | 0.007698 | 0.61 |
| rs13277355 | A | G | 0.274331 | 1.10297 | 0.012996 | 4.7E-14 | -0.00674 | 0.007209 | 0.35 |
| rs143326447 | T | C | 0.881359 | 0.902426 | 0.017883 | 9.4E-09 | -0.01772 | 0.009826 | 0.071 |
| rs150707349 | A | G | 0.96926 | 1.21721 | 0.034805 | 1.6E-08 | 0.025281 | 0.020262 | 0.21 |
| rs1612986 | T | C | 0.816653 | 0.856328 | 0.015037 | 6E-25 | -0.01408 | 0.008307 | 0.09 |
| rs1617333 | A | G | 0.60217 | 1.09812 | 0.011863 | 3E-15 | 0.001798 | 0.006596 | 0.79 |
| rs1655558 | T | G | 0.454064 | 0.919987 | 0.011706 | 1E-12 | 0.001655 | 0.006536 | 0.8 |
| rs16903574 | C | G | 0.924845 | 0.839851 | 0.022396 | 6.5E-15 | 0.005872 | 0.012593 | 0.64 |
| rs17103286 | A | G | 0.478517 | 0.926643 | 0.011603 | 5.2E-11 | 0.004859 | 0.006426 | 0.45 |
| rs17202120 | A | G | 0.982218 | 1.34477 | 0.043928 | 1.5E-11 | -0.033 | 0.024431 | 0.18 |
| rs1806656 | C | G | 0.681098 | 1.08202 | 0.012509 | 2.9E-10 | -0.00383 | 0.007012 | 0.58 |
| rs1837253 | T | C | 0.262925 | 0.846361 | 0.013128 | 5.5E-37 | 0.000781 | 0.007289 | 0.91 |
| rs188074962 | G | A | 0.355239 | 1.11246 | 0.012173 | 2E-18 | -0.00072 | 0.006771 | 0.92 |
| rs1885013 | G | A | 0.281984 | 1.09787 | 0.012849 | 3.7E-13 | 0.009925 | 0.007095 | 0.16 |
| rs1887704 | C | G | 0.32215 | 0.907394 | 0.012388 | 4.3E-15 | 0.002128 | 0.006901 | 0.76 |
| rs1893380 | G | C | 0.6208 | 0.92337 | 0.011913 | 2.2E-11 | -0.00549 | 0.006587 | 0.4 |
| rs1962588 | T | G | 0.791595 | 0.922422 | 0.014396 | 2E-08 | 0.004615 | 0.00807 | 0.57 |
| rs2052690 | G | T | 0.712557 | 0.928894 | 0.012785 | 8E-09 | -0.00289 | 0.007095 | 0.68 |
| rs2066844 | C | T | 0.953476 | 0.853589 | 0.027433 | 7.9E-09 | -0.01859 | 0.014922 | 0.21 |
| rs2070901 | G | T | 0.731764 | 0.914937 | 0.013062 | 1E-11 | 0.002459 | 0.00722 | 0.73 |
| rs2202750 | T | C | 0.381027 | 1.11173 | 0.011896 | 5.4E-19 | 0.007357 | 0.006576 | 0.26 |
| rs2299012 | A | C | 0.808317 | 0.839763 | 0.014686 | 1.3E-32 | -0.00972 | 0.008101 | 0.23 |
| rs230495 | A | G | 0.412214 | 0.936226 | 0.011749 | 2E-08 | -0.00154 | 0.00653 | 0.81 |
| rs2738783 | T | G | 0.212924 | 0.919593 | 0.014227 | 3.8E-09 | -0.0201 | 0.007963 | 0.012 |
| rs274943 | T | C | 0.516893 | 1.07957 | 0.01159 | 4E-11 | 0.00145 | 0.006436 | 0.82 |
| rs2765414 | T | G | 0.225532 | 1.09099 | 0.013821 | 2.9E-10 | 0.003938 | 0.007639 | 0.61 |
| rs2766667 | T | C | 0.256063 | 1.07946 | 0.013289 | 8.7E-09 | 0.01036 | 0.007334 | 0.16 |
| rs2918302 | G | A | 0.846221 | 0.903596 | 0.016049 | 2.7E-10 | 0.005869 | 0.0089 | 0.51 |
| rs2955118 | G | A | 0.725936 | 1.08175 | 0.013029 | 1.6E-09 | 0.016557 | 0.007262 | 0.023 |
| rs3097670 | G | C | 0.878406 | 1.15087 | 0.017894 | 4.1E-15 | 0.010488 | 0.010057 | 0.3 |
| rs3122929 | C | T | 0.600485 | 0.876303 | 0.011843 | 7.2E-29 | 0.001061 | 0.006584 | 0.87 |
| rs340931 | G | A | 0.327612 | 0.905216 | 0.012327 | 6.6E-16 | 0.005622 | 0.006828 | 0.41 |
| rs34290285 | G | A | 0.739377 | 1.19778 | 0.013168 | 9.5E-43 | 0.023574 | 0.00736 | 0.0014 |
| rs35570272 | G | T | 0.604956 | 0.903966 | 0.011885 | 2E-17 | -0.00613 | 0.006602 | 0.35 |
| rs3785356 | C | T | 0.703372 | 0.896086 | 0.012727 | 6.6E-18 | -0.00454 | 0.007072 | 0.52 |
| rs3856439 | C | T | 0.656887 | 1.11341 | 0.012209 | 1.4E-18 | 0.015467 | 0.006818 | 0.023 |
| rs42403 | C | T | 0.887006 | 0.869917 | 0.018294 | 2.6E-14 | 0.024789 | 0.010204 | 0.015 |
| rs4319131 | A | G | 0.544753 | 1.07054 | 0.011651 | 4.9E-09 | 0.016408 | 0.006476 | 0.011 |
| rs45613035 | T | C | 0.903289 | 0.833266 | 0.019553 | 1.1E-20 | -0.01247 | 0.010714 | 0.24 |
| rs4574025 | C | T | 0.465373 | 0.915721 | 0.011658 | 4.3E-14 | 0.00106 | 0.006476 | 0.87 |
| rs4722758 | C | G | 0.800574 | 0.887831 | 0.014435 | 1.7E-16 | 0.016477 | 0.008012 | 0.04 |
| rs4792846 | G | A | 0.459444 | 1.08451 | 0.011645 | 3.2E-12 | 0.01 | 0.00647 | 0.12 |
| rs4795399 | T | C | 0.530853 | 1.30283 | 0.011583 | 1.9E-115 | -0.00186 | 0.006409 | 0.77 |
| rs479844 | A | G | 0.446616 | 0.895698 | 0.011623 | 2.6E-21 | 0.002743 | 0.006435 | 0.67 |
| rs4916534 | A | G | 0.91899 | 1.12664 | 0.021354 | 2.3E-08 | 0.003628 | 0.011979 | 0.76 |
| rs4917131 | A | G | 0.190569 | 1.08637 | 0.014876 | 2.6E-08 | -0.00818 | 0.008336 | 0.33 |
| rs55743914 | C | T | 0.757827 | 0.904782 | 0.013575 | 1.7E-13 | -0.00337 | 0.007541 | 0.66 |
| rs56328339 | C | T | 0.836301 | 1.10129 | 0.015672 | 7.5E-10 | 0.000753 | 0.008714 | 0.93 |
| rs61584523 | G | C | 0.961081 | 1.18425 | 0.030532 | 3E-08 | 0.017389 | 0.017291 | 0.31 |
| rs61815559 | A | T | 0.966971 | 0.633937 | 0.033074 | 3.3E-43 | -0.00181 | 0.018423 | 0.92 |
| rs62408233 | G | A | 0.643957 | 1.11297 | 0.01205 | 6.5E-19 | -0.00075 | 0.006682 | 0.91 |
| rs6927172 | C | G | 0.779472 | 1.08976 | 0.013934 | 6.9E-10 | 0.002248 | 0.007735 | 0.77 |
| rs6954667 | C | A | 0.692969 | 0.931182 | 0.012569 | 1.4E-08 | 0.014321 | 0.006967 | 0.04 |
| rs705700 | T | C | 0.57995 | 0.90236 | 0.011723 | 1.9E-18 | 0.027947 | 0.006497 | 0.000017 |
| rs71421264 | C | G | 0.415631 | 0.925104 | 0.011814 | 4.4E-11 | 0.013451 | 0.006587 | 0.041 |
| rs7209400 | C | T | 0.455041 | 0.924102 | 0.011658 | 1.3E-11 | -0.00024 | 0.006481 | 0.97 |
| rs72743461 | C | A | 0.767112 | 0.836304 | 0.013702 | 6.7E-39 | -0.00748 | 0.007516 | 0.32 |
| rs72823641 | T | A | 0.857876 | 1.34076 | 0.016524 | 1.8E-70 | 0.006463 | 0.009298 | 0.49 |
| rs73192661 | C | T | 0.549501 | 0.881349 | 0.011646 | 2.1E-27 | -0.00752 | 0.00646 | 0.24 |
| rs7582757 | A | G | 0.524664 | 0.937076 | 0.011573 | 2E-08 | 0.001546 | 0.006426 | 0.81 |
| rs7626218 | A | T | 0.602573 | 1.08332 | 0.011823 | 1.3E-11 | 0.017832 | 0.006569 | 0.0066 |
| rs7848215 | C | T | 0.740551 | 0.796999 | 0.013274 | 1.7E-65 | -0.00123 | 0.007393 | 0.87 |
| rs7975763 | C | T | 0.793332 | 1.08807 | 0.014324 | 3.8E-09 | 0.013546 | 0.007993 | 0.09 |
| rs835673 | T | C | 0.265721 | 0.931038 | 0.013093 | 4.8E-08 | -0.00257 | 0.007269 | 0.72 |
| rs892225 | A | G | 0.618623 | 0.925896 | 0.012027 | 1.5E-10 | -0.01566 | 0.006725 | 0.02 |
| rs912425 | G | A | 0.239061 | 1.08376 | 0.01359 | 3.2E-09 | -0.00024 | 0.007535 | 0.97 |
| rs9372120 | T | G | 0.7934 | 0.904475 | 0.014294 | 2.2E-12 | -0.0032 | 0.00789 | 0.68 |

**Table S2** Genetic variants used in the analyses investigating a causal impact of genetically predicted adult-onset asthma and gastrointestinal disorders

| **SNP** | **EA** | **OA** | **EAF** | $\boldsymbol{\beta}$**_expsr_** | **SE_expsr_** | $\boldsymbol{P}$**_expsr_** | $\boldsymbol{\beta}$**_otcm_** | **SE_otcm_** | $\boldsymbol{P}$**_otcm_** |
| --- | --- | --- | --- | --- | --- | --- | --- | --- | --- |
| **adult-onset asthma on IBS** | | | | | | | | | |
| rs1041973 | C | A | 0.770787 | 1.06917 | 0.009862 | 1.2E-11 | 0.004237 | 0.010299 | 0.68 |
| rs1059513 | T | C | 0.891157 | 1.11949 | 0.013352 | 2.8E-17 | -0.01389 | 0.013823 | 0.31 |
| rs11088309 | C | G | 0.858141 | 0.919155 | 0.011921 | 1.5E-12 | 0.014179 | 0.012379 | 0.25 |
| rs11168252 | T | A | 0.759795 | 1.05727 | 0.009774 | 1.2E-08 | -0.00576 | 0.010176 | 0.57 |
| rs11715524 | G | A | 0.490248 | 0.949974 | 0.008366 | 8.5E-10 | -0.00086 | 0.008713 | 0.92 |
| rs11742240 | G | T | 0.722258 | 1.06323 | 0.009291 | 4.1E-11 | -0.00656 | 0.00965 | 0.5 |
| rs117710327 | C | A | 0.931787 | 1.15193 | 0.016863 | 5E-17 | -0.00477 | 0.017985 | 0.79 |
| rs12470864 | G | A | 0.616213 | 0.905753 | 0.008569 | 7.2E-31 | -0.00652 | 0.00884 | 0.46 |
| rs13241235 | T | C | 0.642443 | 1.05041 | 0.008679 | 1.5E-08 | -0.01834 | 0.008966 | 0.041 |
| rs174535 | T | C | 0.648949 | 1.06561 | 0.008722 | 3.2E-13 | 0.003971 | 0.009028 | 0.66 |
| rs17454584 | A | G | 0.7862 | 0.937118 | 0.010138 | 1.5E-10 | -0.01571 | 0.010409 | 0.13 |
| rs17668708 | C | T | 0.892707 | 1.09563 | 0.013551 | 1.6E-11 | -0.0126 | 0.014224 | 0.38 |
| rs1775554 | A | C | 0.573108 | 1.10914 | 0.008413 | 7.8E-35 | 0.01573 | 0.008723 | 0.071 |
| rs1784775 | T | C | 0.291663 | 0.946886 | 0.009155 | 2.5E-09 | -0.0128 | 0.009518 | 0.18 |
| rs1898671 | C | T | 0.652732 | 0.924602 | 0.008728 | 2.7E-19 | -0.00719 | 0.009002 | 0.42 |
| rs2056625 | G | A | 0.589775 | 1.06049 | 0.008449 | 3.6E-12 | 0.008211 | 0.008757 | 0.35 |
| rs2338821 | A | G | 0.384603 | 0.95189 | 0.008555 | 8.2E-09 | -0.00795 | 0.008865 | 0.37 |
| rs2381712 | T | G | 0.479898 | 0.947059 | 0.008327 | 6.5E-11 | -0.01105 | 0.008644 | 0.2 |
| rs28415845 | C | T | 0.700492 | 1.06962 | 0.00914 | 1.8E-13 | -0.0075 | 0.009497 | 0.43 |
| rs28622052 | T | C | 0.976191 | 0.847777 | 0.027287 | 1.4E-09 | -0.05449 | 0.02754 | 0.048 |
| rs28635831 | A | G | 0.649888 | 1.05109 | 0.008761 | 1.3E-08 | -0.00729 | 0.009078 | 0.42 |
| rs301816 | G | A | 0.417313 | 0.953193 | 0.008475 | 1.5E-08 | 0.029391 | 0.00884 | 0.00089 |
| rs3024655 | A | G | 0.061738 | 0.882737 | 0.017371 | 7E-13 | 0.013077 | 0.018123 | 0.47 |
| rs34290285 | G | A | 0.73937 | 1.09969 | 0.009479 | 1.2E-23 | 0.006627 | 0.009872 | 0.5 |
| rs35441874 | T | A | 0.747667 | 1.08093 | 0.00962 | 6E-16 | 0.009507 | 0.010085 | 0.35 |
| rs4099209 | T | G | 0.846519 | 0.917777 | 0.011555 | 1.1E-13 | 0.011903 | 0.011938 | 0.32 |
| rs4491851 | G | A | 0.466068 | 0.946879 | 0.008371 | 7E-11 | 0.003396 | 0.008714 | 0.7 |
| rs4771332 | T | C | 0.316872 | 0.942927 | 0.009001 | 6.6E-11 | -0.00955 | 0.009403 | 0.31 |
| rs4947324 | C | T | 0.897819 | 1.08766 | 0.013707 | 8.8E-10 | -0.02009 | 0.014163 | 0.16 |
| rs57585717 | G | A | 0.881048 | 0.926149 | 0.012843 | 2.3E-09 | 0.008589 | 0.013307 | 0.52 |
| rs62296577 | C | T | 0.668518 | 1.05321 | 0.008849 | 4.7E-09 | 0.008685 | 0.009187 | 0.34 |
| rs66632892 | G | A | 0.740959 | 0.946701 | 0.009554 | 9.9E-09 | -0.01245 | 0.009917 | 0.21 |
| rs6866614 | A | G | 0.426146 | 0.929859 | 0.008487 | 1E-17 | 0.002006 | 0.008813 | 0.82 |
| rs7183955 | A | C | 0.811033 | 1.06955 | 0.010676 | 3E-10 | 0.010806 | 0.011165 | 0.33 |
| rs72743461 | C | A | 0.76717 | 0.911835 | 0.009861 | 8E-21 | -0.00819 | 0.010106 | 0.42 |
| rs7302200 | G | A | 0.662158 | 0.934094 | 0.008813 | 1E-14 | 0.003648 | 0.009119 | 0.69 |
| rs7824278 | C | A | 0.387277 | 1.06233 | 0.008539 | 1.4E-12 | -0.01354 | 0.008875 | 0.13 |
| rs7936312 | G | T | 0.527401 | 0.918629 | 0.008337 | 2.4E-24 | 0.002926 | 0.008622 | 0.73 |
| rs8067124 | A | T | 0.979153 | 0.797016 | 0.032753 | 4.3E-12 | -0.01631 | 0.040957 | 0.69 |
| rs891058 | G | A | 0.70397 | 1.05126 | 0.00912 | 4.2E-08 | 0.001441 | 0.009482 | 0.88 |
| rs943689 | C | T | 0.644135 | 1.07349 | 0.00867 | 2.8E-16 | -0.00293 | 0.008992 | 0.74 |
| rs992969 | A | G | 0.250135 | 1.11548 | 0.00963 | 7.6E-30 | 0.002929 | 0.00992 | 0.77 |
| **adult-onset asthma on IBD** | | | | | | | | | |
| rs1041973 | C | A | 0.770787 | 1.06917 | 0.009862 | 1.2E-11 | -0.01701 | 0.020207 | 0.4 |
| rs1059513 | T | C | 0.891157 | 1.11949 | 0.013352 | 2.8E-17 | 0.039319 | 0.027817 | 0.16 |
| rs11088309 | C | G | 0.858141 | 0.919155 | 0.011921 | 1.5E-12 | 0.009226 | 0.024396 | 0.71 |
| rs11168252 | T | A | 0.759795 | 1.05727 | 0.009774 | 1.2E-08 | -0.04363 | 0.019882 | 0.028 |
| rs11715524 | G | A | 0.490248 | 0.949974 | 0.008366 | 8.5E-10 | -0.0105 | 0.017194 | 0.54 |
| rs11742240 | G | T | 0.722258 | 1.06323 | 0.009291 | 4.1E-11 | -0.02049 | 0.018976 | 0.28 |
| rs117710327 | C | A | 0.931787 | 1.15193 | 0.016863 | 5E-17 | -0.05286 | 0.03476 | 0.13 |
| rs12470864 | G | A | 0.616213 | 0.905753 | 0.008569 | 7.2E-31 | 0.050022 | 0.01755 | 0.0044 |
| rs13241235 | T | C | 0.642443 | 1.05041 | 0.008679 | 1.5E-08 | -0.01724 | 0.017688 | 0.33 |
| rs174535 | T | C | 0.648949 | 1.06561 | 0.008722 | 3.2E-13 | -0.00537 | 0.017787 | 0.76 |
| rs17454584 | A | G | 0.7862 | 0.937118 | 0.010138 | 1.5E-10 | -0.06906 | 0.020233 | 0.00064 |
| rs17668708 | C | T | 0.892707 | 1.09563 | 0.013551 | 1.6E-11 | -0.05987 | 0.02754 | 0.03 |
| rs1775554 | A | C | 0.573108 | 1.10914 | 0.008413 | 7.8E-35 | 0.015094 | 0.01721 | 0.38 |
| rs1784775 | T | C | 0.291663 | 0.946886 | 0.009155 | 2.5E-09 | 0.013876 | 0.018679 | 0.46 |
| rs1898671 | C | T | 0.652732 | 0.924602 | 0.008728 | 2.7E-19 | -0.0169 | 0.017733 | 0.34 |
| rs2056625 | G | A | 0.589775 | 1.06049 | 0.008449 | 3.6E-12 | 0.001805 | 0.017266 | 0.92 |
| rs2338821 | A | G | 0.384603 | 0.95189 | 0.008555 | 8.2E-09 | -0.0244 | 0.017523 | 0.16 |
| rs2381712 | T | G | 0.479898 | 0.947059 | 0.008327 | 6.5E-11 | -0.00017 | 0.017051 | 0.99 |
| rs28415845 | C | T | 0.700492 | 1.06962 | 0.00914 | 1.8E-13 | -0.00817 | 0.018731 | 0.66 |
| rs28622052 | T | C | 0.976191 | 0.847777 | 0.027287 | 1.4E-09 | 0.101431 | 0.058227 | 0.082 |
| rs28635831 | A | G | 0.649888 | 1.05109 | 0.008761 | 1.3E-08 | 0.02945 | 0.018008 | 0.1 |
| rs301816 | G | A | 0.417313 | 0.953193 | 0.008475 | 1.5E-08 | 0.031336 | 0.017431 | 0.072 |
| rs3024655 | A | G | 0.061738 | 0.882737 | 0.017371 | 7E-13 | -0.03131 | 0.036421 | 0.39 |
| rs34290285 | G | A | 0.73937 | 1.09969 | 0.009479 | 1.2E-23 | -0.04317 | 0.019255 | 0.025 |
| rs35441874 | T | A | 0.747667 | 1.08093 | 0.00962 | 6E-16 | 0.001913 | 0.019865 | 0.92 |
| rs4099209 | T | G | 0.846519 | 0.917777 | 0.011555 | 1.1E-13 | -0.00638 | 0.023417 | 0.79 |
| rs4491851 | G | A | 0.466068 | 0.946879 | 0.008371 | 7E-11 | 0.01829 | 0.017184 | 0.29 |
| rs4771332 | T | C | 0.316872 | 0.942927 | 0.009001 | 6.6E-11 | 0.030181 | 0.018423 | 0.1 |
| rs4947324 | C | T | 0.897819 | 1.08766 | 0.013707 | 8.8E-10 | 0.079062 | 0.029011 | 0.0064 |
| rs57585717 | G | A | 0.881048 | 0.926149 | 0.012843 | 2.3E-09 | 0.02092 | 0.026378 | 0.43 |
| rs62296577 | C | T | 0.668518 | 1.05321 | 0.008849 | 4.7E-09 | 0.001302 | 0.018107 | 0.94 |
| rs66632892 | G | A | 0.740959 | 0.946701 | 0.009554 | 9.9E-09 | -0.0081 | 0.019579 | 0.68 |
| rs6866614 | A | G | 0.426146 | 0.929859 | 0.008487 | 1E-17 | 0.082125 | 0.017291 | 0.000002 |
| rs7183955 | A | C | 0.811033 | 1.06955 | 0.010676 | 3E-10 | 0.038171 | 0.022222 | 0.086 |
| rs72743461 | C | A | 0.76717 | 0.911835 | 0.009861 | 8E-21 | -0.0848 | 0.019552 | 0.000014 |
| rs7302200 | G | A | 0.662158 | 0.934094 | 0.008813 | 1E-14 | 0.005567 | 0.017997 | 0.76 |
| rs7824278 | C | A | 0.387277 | 1.06233 | 0.008539 | 1.4E-12 | -0.05796 | 0.017599 | 0.00099 |
| rs7936312 | G | T | 0.527401 | 0.918629 | 0.008337 | 2.4E-24 | -0.11086 | 0.016983 | 6.7E-11 |
| rs8067124 | A | T | 0.979153 | 0.797016 | 0.032753 | 4.3E-12 | -0.20187 | 0.073803 | 0.0062 |
| rs891058 | G | A | 0.70397 | 1.05126 | 0.00912 | 4.2E-08 | -0.02405 | 0.018613 | 0.2 |
| rs943689 | C | T | 0.644135 | 1.07349 | 0.00867 | 2.8E-16 | 0.043807 | 0.017858 | 0.014 |
| rs992969 | A | G | 0.250135 | 1.11548 | 0.00963 | 7.6E-30 | 0.027719 | 0.019455 | 0.15 |
| **adult-onset asthma on PUD** | | | | | | | | | |
| rs1041973 | C | A | 0.770787 | 1.06917 | 0.009862 | 1.2E-11 | 0.003052 | 0.013297 | 0.82 |
| rs1059513 | T | C | 0.891157 | 1.11949 | 0.013352 | 2.8E-17 | 0.007025 | 0.017983 | 0.7 |
| rs11088309 | C | G | 0.858141 | 0.919155 | 0.011921 | 1.5E-12 | 0.009736 | 0.015968 | 0.54 |
| rs11168252 | T | A | 0.759795 | 1.05727 | 0.009774 | 1.2E-08 | -0.00338 | 0.013147 | 0.8 |
| rs11715524 | G | A | 0.490248 | 0.949974 | 0.008366 | 8.5E-10 | 0.005462 | 0.011252 | 0.63 |
| rs11742240 | G | T | 0.722258 | 1.06323 | 0.009291 | 4.1E-11 | 0.00348 | 0.012487 | 0.78 |
| rs117710327 | C | A | 0.931787 | 1.15193 | 0.016863 | 5E-17 | -0.05195 | 0.022783 | 0.023 |
| rs12470864 | G | A | 0.616213 | 0.905753 | 0.008569 | 7.2E-31 | -0.0081 | 0.011413 | 0.48 |
| rs13241235 | T | C | 0.642443 | 1.05041 | 0.008679 | 1.5E-08 | 0.004296 | 0.011612 | 0.71 |
| rs174535 | T | C | 0.648949 | 1.06561 | 0.008722 | 3.2E-13 | -0.03334 | 0.011599 | 0.004 |
| rs17454584 | A | G | 0.7862 | 0.937118 | 0.010138 | 1.5E-10 | 0.006553 | 0.013517 | 0.63 |
| rs17668708 | C | T | 0.892707 | 1.09563 | 0.013551 | 1.6E-11 | -0.02537 | 0.018278 | 0.17 |
| rs1775554 | A | C | 0.573108 | 1.10914 | 0.008413 | 7.8E-35 | 0.007457 | 0.011258 | 0.51 |
| rs1784775 | T | C | 0.291663 | 0.946886 | 0.009155 | 2.5E-09 | -0.03797 | 0.012354 | 0.0021 |
| rs1898671 | C | T | 0.652732 | 0.924602 | 0.008728 | 2.7E-19 | 0.002648 | 0.01164 | 0.82 |
| rs2056625 | G | A | 0.589775 | 1.06049 | 0.008449 | 3.6E-12 | -0.0203 | 0.011282 | 0.072 |
| rs2338821 | A | G | 0.384603 | 0.95189 | 0.008555 | 8.2E-09 | -0.01101 | 0.011452 | 0.34 |
| rs2381712 | T | G | 0.479898 | 0.947059 | 0.008327 | 6.5E-11 | -0.01746 | 0.011165 | 0.12 |
| rs28415845 | C | T | 0.700492 | 1.06962 | 0.00914 | 1.8E-13 | -0.00446 | 0.01227 | 0.72 |
| rs28622052 | T | C | 0.976191 | 0.847777 | 0.027287 | 1.4E-09 | -0.00572 | 0.036289 | 0.87 |
| rs28635831 | A | G | 0.649888 | 1.05109 | 0.008761 | 1.3E-08 | -0.00339 | 0.01173 | 0.77 |
| rs301816 | G | A | 0.417313 | 0.953193 | 0.008475 | 1.5E-08 | 0.013944 | 0.011428 | 0.22 |
| rs3024655 | A | G | 0.061738 | 0.882737 | 0.017371 | 7E-13 | -0.02617 | 0.023776 | 0.27 |
| rs34290285 | G | A | 0.73937 | 1.09969 | 0.009479 | 1.2E-23 | 0.010916 | 0.012763 | 0.39 |
| rs35441874 | T | A | 0.747667 | 1.08093 | 0.00962 | 6E-16 | -0.0044 | 0.012983 | 0.73 |
| rs4099209 | T | G | 0.846519 | 0.917777 | 0.011555 | 1.1E-13 | 0.010067 | 0.015411 | 0.51 |
| rs4491851 | G | A | 0.466068 | 0.946879 | 0.008371 | 7E-11 | -0.00633 | 0.011256 | 0.57 |
| rs4771332 | T | C | 0.316872 | 0.942927 | 0.009001 | 6.6E-11 | -0.00896 | 0.012143 | 0.46 |
| rs4947324 | C | T | 0.897819 | 1.08766 | 0.013707 | 8.8E-10 | -0.03144 | 0.018204 | 0.084 |
| rs57585717 | G | A | 0.881048 | 0.926149 | 0.012843 | 2.3E-09 | 0.005767 | 0.017168 | 0.74 |
| rs62296577 | C | T | 0.668518 | 1.05321 | 0.008849 | 4.7E-09 | -0.01524 | 0.01182 | 0.2 |
| rs66632892 | G | A | 0.740959 | 0.946701 | 0.009554 | 9.9E-09 | 0.000554 | 0.012841 | 0.97 |
| rs6866614 | A | G | 0.426146 | 0.929859 | 0.008487 | 1E-17 | -0.02226 | 0.0114 | 0.051 |
| rs7183955 | A | C | 0.811033 | 1.06955 | 0.010676 | 3E-10 | 0.016069 | 0.014443 | 0.27 |
| rs72743461 | C | A | 0.76717 | 0.911835 | 0.009861 | 8E-21 | 0.011965 | 0.013111 | 0.36 |
| rs7302200 | G | A | 0.662158 | 0.934094 | 0.008813 | 1E-14 | 0.00923 | 0.011786 | 0.43 |
| rs7824278 | C | A | 0.387277 | 1.06233 | 0.008539 | 1.4E-12 | 0.019947 | 0.011421 | 0.081 |
| rs7936312 | G | T | 0.527401 | 0.918629 | 0.008337 | 2.4E-24 | -0.00655 | 0.011131 | 0.56 |
| rs8067124 | A | T | 0.979153 | 0.797016 | 0.032753 | 4.3E-12 | -0.02272 | 0.052669 | 0.67 |
| rs891058 | G | A | 0.70397 | 1.05126 | 0.00912 | 4.2E-08 | -0.01373 | 0.01221 | 0.26 |
| rs943689 | C | T | 0.644135 | 1.07349 | 0.00867 | 2.8E-16 | 0.006055 | 0.011625 | 0.6 |
| rs992969 | A | G | 0.250135 | 1.11548 | 0.00963 | 7.6E-30 | -0.0115 | 0.012853 | 0.37 |
| **adult-onset asthma on GORD** | | | | | | | | | |
| rs1041973 | C | A | 0.770787 | 1.06917 | 0.009862 | 1.2E-11 | 0.014093 | 0.00767 | 0.066 |
| rs1059513 | T | C | 0.891157 | 1.11949 | 0.013352 | 2.8E-17 | -0.00688 | 0.010306 | 0.5 |
| rs11088309 | C | G | 0.858141 | 0.919155 | 0.011921 | 1.5E-12 | 0.001921 | 0.009171 | 0.83 |
| rs11168252 | T | A | 0.759795 | 1.05727 | 0.009774 | 1.2E-08 | 0.023508 | 0.007611 | 0.002 |
| rs11715524 | G | A | 0.490248 | 0.949974 | 0.008366 | 8.5E-10 | -0.00973 | 0.006478 | 0.13 |
| rs11742240 | G | T | 0.722258 | 1.06323 | 0.009291 | 4.1E-11 | -0.01064 | 0.00717 | 0.14 |
| rs117710327 | C | A | 0.931787 | 1.15193 | 0.016863 | 5E-17 | 0.011031 | 0.013447 | 0.41 |
| rs12470864 | G | A | 0.616213 | 0.905753 | 0.008569 | 7.2E-31 | 0.008676 | 0.006579 | 0.19 |
| rs13241235 | T | C | 0.642443 | 1.05041 | 0.008679 | 1.5E-08 | -0.00618 | 0.006677 | 0.35 |
| rs174535 | T | C | 0.648949 | 1.06561 | 0.008722 | 3.2E-13 | -0.00431 | 0.006705 | 0.52 |
| rs17454584 | A | G | 0.7862 | 0.937118 | 0.010138 | 1.5E-10 | -0.00467 | 0.007761 | 0.55 |
| rs17668708 | C | T | 0.892707 | 1.09563 | 0.013551 | 1.6E-11 | 0.002302 | 0.010628 | 0.83 |
| rs1775554 | A | C | 0.573108 | 1.10914 | 0.008413 | 7.8E-35 | 0.013029 | 0.006483 | 0.044 |
| rs1784775 | T | C | 0.291663 | 0.946886 | 0.009155 | 2.5E-09 | -0.0104 | 0.007071 | 0.14 |
| rs1898671 | C | T | 0.652732 | 0.924602 | 0.008728 | 2.7E-19 | 0.012767 | 0.006708 | 0.057 |
| rs2056625 | G | A | 0.589775 | 1.06049 | 0.008449 | 3.6E-12 | 0.001145 | 0.006506 | 0.86 |
| rs2338821 | A | G | 0.384603 | 0.95189 | 0.008555 | 8.2E-09 | -0.01174 | 0.006591 | 0.075 |
| rs2381712 | T | G | 0.479898 | 0.947059 | 0.008327 | 6.5E-11 | -0.00249 | 0.006424 | 0.7 |
| rs28415845 | C | T | 0.700492 | 1.06962 | 0.00914 | 1.8E-13 | -0.00195 | 0.007068 | 0.78 |
| rs28622052 | T | C | 0.976191 | 0.847777 | 0.027287 | 1.4E-09 | 0.002426 | 0.020963 | 0.91 |
| rs28635831 | A | G | 0.649888 | 1.05109 | 0.008761 | 1.3E-08 | 0.010857 | 0.006766 | 0.11 |
| rs301816 | G | A | 0.417313 | 0.953193 | 0.008475 | 1.5E-08 | 0.014566 | 0.006579 | 0.027 |
| rs3024655 | A | G | 0.061738 | 0.882737 | 0.017371 | 7E-13 | -0.00285 | 0.013557 | 0.83 |
| rs34290285 | G | A | 0.73937 | 1.09969 | 0.009479 | 1.2E-23 | 0.023574 | 0.00736 | 0.0014 |
| rs35441874 | T | A | 0.747667 | 1.08093 | 0.00962 | 6E-16 | -0.00314 | 0.007479 | 0.67 |
| rs4099209 | T | G | 0.846519 | 0.917777 | 0.011555 | 1.1E-13 | -0.0036 | 0.008834 | 0.68 |
| rs4491851 | G | A | 0.466068 | 0.946879 | 0.008371 | 7E-11 | -0.0166 | 0.006482 | 0.01 |
| rs4771332 | T | C | 0.316872 | 0.942927 | 0.009001 | 6.6E-11 | 0.003857 | 0.006977 | 0.58 |
| rs4947324 | C | T | 0.897819 | 1.08766 | 0.013707 | 8.8E-10 | -0.03871 | 0.01048 | 0.00022 |
| rs57585717 | G | A | 0.881048 | 0.926149 | 0.012843 | 2.3E-09 | 0.02172 | 0.009926 | 0.029 |
| rs62296577 | C | T | 0.668518 | 1.05321 | 0.008849 | 4.7E-09 | 0.022831 | 0.006841 | 0.00085 |
| rs66632892 | G | A | 0.740959 | 0.946701 | 0.009554 | 9.9E-09 | -0.00822 | 0.007382 | 0.27 |
| rs6866614 | A | G | 0.426146 | 0.929859 | 0.008487 | 1E-17 | -0.01309 | 0.006557 | 0.046 |
| rs7183955 | A | C | 0.811033 | 1.06955 | 0.010676 | 3E-10 | 0.002197 | 0.008282 | 0.79 |
| rs72743461 | C | A | 0.76717 | 0.911835 | 0.009861 | 8E-21 | -0.00748 | 0.007516 | 0.32 |
| rs7302200 | G | A | 0.662158 | 0.934094 | 0.008813 | 1E-14 | 0.022952 | 0.006795 | 0.00073 |
| rs7824278 | C | A | 0.387277 | 1.06233 | 0.008539 | 1.4E-12 | 0.007357 | 0.006585 | 0.26 |
| rs7936312 | G | T | 0.527401 | 0.918629 | 0.008337 | 2.4E-24 | 0.002738 | 0.00641 | 0.67 |
| rs8067124 | A | T | 0.979153 | 0.797016 | 0.032753 | 4.3E-12 | 0.008043 | 0.030732 | 0.79 |
| rs891058 | G | A | 0.70397 | 1.05126 | 0.00912 | 4.2E-08 | 0.014619 | 0.007062 | 0.038 |
| rs943689 | C | T | 0.644135 | 1.07349 | 0.00867 | 2.8E-16 | -6.9E-05 | 0.006687 | 0.99 |
| rs992969 | A | G | 0.250135 | 1.11548 | 0.00963 | 7.6E-30 | 0.008082 | 0.00737 | 0.27 |

**Table S3** Genetic variants used in the analyses investigating a causal impact of genetically predicted childhood-onset asthma and inflammatory bowel disease (IBD) including its subgroups Crohn´s disease (CD) and ulcerative colitis (UC)

| **SNP** | **EA** | **OA** | **EAF** | $\boldsymbol{\beta}$**_expsr_** | **SE_expsr_** | $\boldsymbol{P}$**_expsr_** | $\boldsymbol{\beta}$**_otcm_** | **SE_otcm_** | $\boldsymbol{P}$**_otcm_** |
| --- | --- | --- | --- | --- | --- | --- | --- | --- | --- |
| **childhood-onset asthma on IBD** | | | | | | | | | |
| rs10118244 | C | T | 0.971414 | 1.21258 | 0.034716 | 2.8E-08 | 0.101103 | 0.049367 | 0.04056 |
| rs10158467 | A | G | 0.714141 | 0.896019 | 0.012885 | 1.6E-17 | -0.04 | 0.018727 | 0.03269 |
| rs10786050 | A | G | 0.406743 | 0.917752 | 0.011754 | 2.8E-13 | -0.0501 | 0.017099 | 0.003386 |
| rs10836538 | G | T | 0.651655 | 1.08321 | 0.012173 | 5.2E-11 | 0.029604 | 0.017618 | 0.09289 |
| rs10995245 | G | A | 0.652823 | 0.931956 | 0.012168 | 7E-09 | 0.026899 | 0.017719 | 0.129 |
| rs11065979 | C | T | 0.558799 | 1.08502 | 0.011655 | 2.5E-12 | -0.0665 | 0.01737 | 0.000129 |
| rs11071559 | C | T | 0.868457 | 1.18496 | 0.017078 | 2.9E-23 | 0.099003 | 0.025084 | 7.92E-05 |
| rs11135015 | T | C | 0.670957 | 0.934697 | 0.012296 | 4E-08 | -0.0663 | 0.018567 | 0.000356 |
| rs11236797 | C | A | 0.552697 | 0.823406 | 0.011634 | 1.3E-62 | -0.1557 | 0.016981 | 4.75E-20 |
| rs11593589 | C | A | 0.457859 | 1.07221 | 0.011592 | 1.8E-09 | -0.0812 | 0.017309 | 2.71E-06 |
| rs11658582 | C | G | 0.379145 | 0.912738 | 0.011907 | 1.7E-14 | -0.0247 | 0.017341 | 0.1543 |
| rs12023876 | G | T | 0.667519 | 1.07987 | 0.01226 | 3.7E-10 | -0.0169 | 0.017948 | 0.3465 |
| rs12365699 | G | A | 0.831274 | 1.14126 | 0.015499 | 1.5E-17 | 0.015103 | 0.023093 | 0.5131 |
| rs12531500 | A | G | 0.565634 | 1.10309 | 0.011689 | 4.7E-17 | -0.0112 | 0.016995 | 0.5098 |
| rs12657787 | G | A | 0.847423 | 0.849237 | 0.01615 | 4.6E-24 | -0.041 | 0.022857 | 0.07286 |
| rs12935657 | G | A | 0.749187 | 1.15436 | 0.013371 | 6.9E-27 | 0.011698 | 0.019052 | 0.5392 |
| rs13277355 | A | G | 0.274331 | 1.10297 | 0.012996 | 4.7E-14 | -0.0212 | 0.019319 | 0.2724 |
| rs1612986 | T | C | 0.816653 | 0.856328 | 0.015037 | 6E-25 | -0.0313 | 0.021699 | 0.1491 |
| rs1617333 | A | G | 0.60217 | 1.09812 | 0.011863 | 3E-15 | -0.006 | 0.018271 | 0.7427 |
| rs1655558 | T | G | 0.454064 | 0.919987 | 0.011706 | 1E-12 | 0.054801 | 0.017498 | 0.001737 |
| rs16903574 | C | G | 0.924845 | 0.839851 | 0.022396 | 6.5E-15 | 0.006896 | 0.033942 | 0.839 |
| rs17103286 | A | G | 0.478517 | 0.926643 | 0.011603 | 5.2E-11 | -0.0459 | 0.01683 | 0.00639 |
| rs1806656 | C | G | 0.681098 | 1.08202 | 0.012509 | 2.9E-10 | -0.0018 | 0.01864 | 0.923 |
| rs1837253 | T | C | 0.262925 | 0.846361 | 0.013128 | 5.5E-37 | -0.0273 | 0.018931 | 0.1493 |
| rs1885013 | G | A | 0.281984 | 1.09787 | 0.012849 | 3.7E-13 | -0.0349 | 0.018321 | 0.05676 |
| rs1887704 | C | G | 0.32215 | 0.907394 | 0.012388 | 4.3E-15 | -0.0072 | 0.017656 | 0.6836 |
| rs1893380 | G | C | 0.6208 | 0.92337 | 0.011913 | 2.2E-11 | -0.0497 | 0.017205 | 0.003865 |
| rs1962588 | T | G | 0.791595 | 0.922422 | 0.014396 | 2E-08 | -0.0166 | 0.021908 | 0.4487 |
| rs2052690 | G | T | 0.712557 | 0.928894 | 0.012785 | 8E-09 | 0.006199 | 0.018614 | 0.7391 |
| rs2066844 | C | T | 0.953476 | 0.853589 | 0.027433 | 7.9E-09 | -0.3107 | 0.037329 | 8.55E-17 |
| rs2202750 | T | C | 0.381027 | 1.11173 | 0.011896 | 5.4E-19 | -0.0456 | 0.017115 | 0.007707 |
| rs2299012 | A | C | 0.808317 | 0.839763 | 0.014686 | 1.3E-32 | 0.016601 | 0.02081 | 0.425 |
| rs230495 | A | G | 0.412214 | 0.936226 | 0.011749 | 2E-08 | 0.023101 | 0.017099 | 0.1767 |
| rs2738783 | T | G | 0.212924 | 0.919593 | 0.014227 | 3.8E-09 | -0.194 | 0.024369 | 1.71E-15 |
| rs274943 | T | C | 0.516893 | 1.07957 | 0.01159 | 4E-11 | -0.02 | 0.016811 | 0.2342 |
| rs2765414 | T | G | 0.225532 | 1.09099 | 0.013821 | 2.9E-10 | 0.028704 | 0.019739 | 0.1459 |
| rs2766667 | T | C | 0.256063 | 1.07946 | 0.013289 | 8.7E-09 | -0.0049 | 0.019478 | 0.8013 |
| rs2918302 | G | A | 0.846221 | 0.903596 | 0.016049 | 2.7E-10 | -0.0586 | 0.023383 | 0.01221 |
| rs2955118 | G | A | 0.725936 | 1.08175 | 0.013029 | 1.6E-09 | -0.0013 | 0.018299 | 0.9434 |
| rs3122929 | C | T | 0.600485 | 0.876303 | 0.011843 | 7.2E-29 | 0.001501 | 0.017438 | 0.9314 |
| rs340931 | G | A | 0.327612 | 0.905216 | 0.012327 | 6.6E-16 | -0.0091 | 0.017883 | 0.6109 |
| rs35570272 | G | T | 0.604956 | 0.903966 | 0.011885 | 2E-17 | -0.0147 | 0.01735 | 0.3968 |
| rs3785356 | C | T | 0.703372 | 0.896086 | 0.012727 | 6.6E-18 | -0.0068 | 0.018478 | 0.713 |
| rs3856439 | C | T | 0.656887 | 1.11341 | 0.012209 | 1.4E-18 | -0.032 | 0.017992 | 0.07529 |
| rs42403 | C | T | 0.887006 | 0.869917 | 0.018294 | 2.6E-14 | 0.065499 | 0.026557 | 0.01365 |
| rs4319131 | A | G | 0.544753 | 1.07054 | 0.011651 | 4.9E-09 | 0.016798 | 0.016888 | 0.3199 |
| rs45613035 | T | C | 0.903289 | 0.833266 | 0.019553 | 1.1E-20 | -0.089 | 0.030912 | 0.003989 |
| rs4574025 | C | T | 0.465373 | 0.915721 | 0.011658 | 4.3E-14 | 0.019101 | 0.017306 | 0.2697 |
| rs4722758 | C | G | 0.800574 | 0.887831 | 0.014435 | 1.7E-16 | -0.027 | 0.020282 | 0.1831 |
| rs4792846 | G | A | 0.459444 | 1.08451 | 0.011645 | 3.2E-12 | 0.021499 | 0.017689 | 0.2242 |
| rs4795399 | T | C | 0.530853 | 1.30283 | 0.011583 | 1.9E-115 | -0.1374 | 0.016757 | 2.41E-16 |
| rs479844 | A | G | 0.446616 | 0.895698 | 0.011623 | 2.6E-21 | 0.018596 | 0.01683 | 0.2692 |
| rs4916534 | A | G | 0.91899 | 1.12664 | 0.021354 | 2.3E-08 | -0.0229 | 0.034473 | 0.5065 |
| rs4917131 | A | G | 0.190569 | 1.08637 | 0.014876 | 2.6E-08 | -0.0958 | 0.022324 | 1.78E-05 |
| rs55743914 | C | T | 0.757827 | 0.904782 | 0.013575 | 1.7E-13 | 0.015103 | 0.02031 | 0.4571 |
| rs56328339 | C | T | 0.836301 | 1.10129 | 0.015672 | 7.5E-10 | 0.082295 | 0.024133 | 0.00065 |
| rs62408233 | G | A | 0.643957 | 1.11297 | 0.01205 | 6.5E-19 | 0.087597 | 0.017693 | 7.39E-07 |
| rs6846348 | G | A | 0.686305 | 1.08507 | 0.012659 | 1.1E-10 | 0.030799 | 0.017888 | 0.0851 |
| rs6927172 | C | G | 0.779472 | 1.08976 | 0.013934 | 6.9E-10 | -0.11029 | 0.020184 | 4.65E-08 |
| rs6954667 | C | A | 0.692969 | 0.931182 | 0.012569 | 1.4E-08 | 0.006702 | 0.018803 | 0.7215 |
| rs705700 | T | C | 0.57995 | 0.90236 | 0.011723 | 1.9E-18 | 0.011504 | 0.017099 | 0.5011 |
| rs7209400 | C | T | 0.455041 | 0.924102 | 0.011658 | 1.3E-11 | 0.008496 | 0.016793 | 0.6129 |
| rs72743461 | C | A | 0.767112 | 0.836304 | 0.013702 | 6.7E-39 | -0.1491 | 0.019868 | 6.17E-14 |
| rs72823641 | T | A | 0.857876 | 1.34076 | 0.016524 | 1.8E-70 | 0.018205 | 0.024062 | 0.4493 |
| rs73192661 | C | T | 0.549501 | 0.881349 | 0.011646 | 2.1E-27 | 0.014403 | 0.016973 | 0.3961 |
| rs7582757 | A | G | 0.524664 | 0.937076 | 0.011573 | 2E-08 | -0.0315 | 0.016819 | 0.06107 |
| rs7625643 | A | G | 0.556922 | 0.934908 | 0.011791 | 1.1E-08 | -0.0323 | 0.01729 | 0.06178 |
| rs7626218 | A | T | 0.602573 | 1.08332 | 0.011823 | 1.3E-11 | -0.0042 | 0.017178 | 0.8069 |
| rs7848215 | C | T | 0.740551 | 0.796999 | 0.013274 | 1.7E-65 | -0.025 | 0.019058 | 0.1895 |
| rs7975763 | C | T | 0.793332 | 1.08807 | 0.014324 | 3.8E-09 | -0.0139 | 0.020455 | 0.4967 |
| rs835673 | T | C | 0.265721 | 0.931038 | 0.013093 | 4.8E-08 | 0.049504 | 0.018869 | 0.008703 |
| rs912425 | G | A | 0.239061 | 1.08376 | 0.01359 | 3.2E-09 | 0.0439 | 0.019391 | 0.02358 |
| rs9372120 | T | G | 0.7934 | 0.904475 | 0.014294 | 2.2E-12 | 0.020205 | 0.021263 | 0.342 |
| rs9391997 | A | G | 0.469864 | 0.928512 | 0.011564 | 1.4E-10 | -0.0013 | 0.017225 | 0.9398 |
| rs9662290 | T | C | 0.672699 | 0.932691 | 0.012669 | 3.8E-08 | -0.0142 | 0.018978 | 0.4543 |
| **childhood-onset asthma on CD** | | | | | | | | | |
| rs10118244 | C | T | 0.971414 | 1.21258 | 0.034716 | 2.8E-08 | 0.1513 | 0.069636 | 0.0298 |
| rs10158467 | A | G | 0.714141 | 0.896019 | 0.012885 | 1.6E-17 | -0.0459 | 0.025371 | 0.07044 |
| rs10187276 | T | C | 0.251175 | 1.11605 | 0.013347 | 1.9E-16 | -0.0458 | 0.029173 | 0.1164 |
| rs10786050 | A | G | 0.406743 | 0.917752 | 0.011754 | 2.8E-13 | -0.046 | 0.023325 | 0.04858 |
| rs10836538 | G | T | 0.651655 | 1.08321 | 0.012173 | 5.2E-11 | 0.042396 | 0.024048 | 0.07791 |
| rs10995245 | G | A | 0.652823 | 0.931956 | 0.012168 | 7E-09 | 0.040499 | 0.024231 | 0.09465 |
| rs11065979 | C | T | 0.558799 | 1.08502 | 0.011655 | 2.5E-12 | -0.0739 | 0.02377 | 0.001877 |
| rs11071559 | C | T | 0.868457 | 1.18496 | 0.017078 | 2.9E-23 | 0.0689 | 0.034134 | 0.04354 |
| rs11135015 | T | C | 0.670957 | 0.934697 | 0.012296 | 4E-08 | -0.0418 | 0.02523 | 0.09756 |
| rs11236797 | C | A | 0.552697 | 0.823406 | 0.011634 | 1.3E-62 | -0.1811 | 0.023128 | 4.85E-15 |
| rs11593589 | C | A | 0.457859 | 1.07221 | 0.011592 | 1.8E-09 | -0.0701 | 0.02359 | 0.002961 |
| rs11658582 | C | G | 0.379145 | 0.912738 | 0.011907 | 1.7E-14 | 0.015204 | 0.023695 | 0.5211 |
| rs12023876 | G | T | 0.667519 | 1.07987 | 0.01226 | 3.7E-10 | 0.001902 | 0.024061 | 0.937 |
| rs12365699 | G | A | 0.831274 | 1.14126 | 0.015499 | 1.5E-17 | 0.043398 | 0.031597 | 0.1696 |
| rs12531500 | A | G | 0.565634 | 1.10309 | 0.011689 | 4.7E-17 | -0.0039 | 0.02284 | 0.8645 |
| rs12657787 | G | A | 0.847423 | 0.849237 | 0.01615 | 4.6E-24 | 0.009101 | 0.031157 | 0.7702 |
| rs12935657 | G | A | 0.749187 | 1.15436 | 0.013371 | 6.9E-27 | -0.0248 | 0.025628 | 0.3332 |
| rs13277355 | A | G | 0.274331 | 1.10297 | 0.012996 | 4.7E-14 | 0.0446 | 0.025718 | 0.08288 |
| rs1612986 | T | C | 0.816653 | 0.856328 | 0.015037 | 6E-25 | -0.0525 | 0.029611 | 0.0762 |
| rs1617333 | A | G | 0.60217 | 1.09812 | 0.011863 | 3E-15 | -0.0441 | 0.025193 | 0.08005 |
| rs1655558 | T | G | 0.454064 | 0.919987 | 0.011706 | 1E-12 | 0.022202 | 0.023775 | 0.3504 |
| rs17103286 | A | G | 0.478517 | 0.926643 | 0.011603 | 5.2E-11 | -0.0457 | 0.023036 | 0.04728 |
| rs1806656 | C | G | 0.681098 | 1.08202 | 0.012509 | 2.9E-10 | -0.0162 | 0.025691 | 0.5283 |
| rs1837253 | T | C | 0.262925 | 0.846361 | 0.013128 | 5.5E-37 | -0.0184 | 0.025685 | 0.4738 |
| rs1885013 | G | A | 0.281984 | 1.09787 | 0.012849 | 3.7E-13 | -0.0226 | 0.024915 | 0.3643 |
| rs1887704 | C | G | 0.32215 | 0.907394 | 0.012388 | 4.3E-15 | -0.0155 | 0.024086 | 0.5199 |
| rs1893380 | G | C | 0.6208 | 0.92337 | 0.011913 | 2.2E-11 | -0.0356 | 0.023475 | 0.1294 |
| rs1962588 | T | G | 0.791595 | 0.922422 | 0.014396 | 2E-08 | -0.0716 | 0.030389 | 0.01846 |
| rs2052690 | G | T | 0.712557 | 0.928894 | 0.012785 | 8E-09 | 0.0571 | 0.025335 | 0.02421 |
| rs2066844 | C | T | 0.953476 | 0.853589 | 0.027433 | 7.9E-09 | -0.6274 | 0.0445 | 3.86E-45 |
| rs2070901 | G | T | 0.731764 | 0.914937 | 0.013062 | 1E-11 | -0.0094 | 0.025845 | 0.7162 |
| rs2202750 | T | C | 0.381027 | 1.11173 | 0.011896 | 5.4E-19 | -0.0219 | 0.023107 | 0.3433 |
| rs2299012 | A | C | 0.808317 | 0.839763 | 0.014686 | 1.3E-32 | 0.034102 | 0.028385 | 0.2296 |
| rs230495 | A | G | 0.412214 | 0.936226 | 0.011749 | 2E-08 | -0.0444 | 0.023576 | 0.05966 |
| rs2738783 | T | G | 0.212924 | 0.919593 | 0.014227 | 3.8E-09 | -0.19171 | 0.033853 | 1.49E-08 |
| rs274943 | T | C | 0.516893 | 1.07957 | 0.01159 | 4E-11 | -0.0235 | 0.022923 | 0.3052 |
| rs2765414 | T | G | 0.225532 | 1.09099 | 0.013821 | 2.9E-10 | 0.021204 | 0.026676 | 0.4267 |
| rs2766667 | T | C | 0.256063 | 1.07946 | 0.013289 | 8.7E-09 | 0.014701 | 0.026385 | 0.5774 |
| rs2918302 | G | A | 0.846221 | 0.903596 | 0.016049 | 2.7E-10 | -0.0312 | 0.032528 | 0.3375 |
| rs2955118 | G | A | 0.725936 | 1.08175 | 0.013029 | 1.6E-09 | 0.032596 | 0.025451 | 0.2003 |
| rs3122929 | C | T | 0.600485 | 0.876303 | 0.011843 | 7.2E-29 | 0.031697 | 0.024344 | 0.1929 |
| rs340931 | G | A | 0.327612 | 0.905216 | 0.012327 | 6.6E-16 | -0.0332 | 0.024259 | 0.1711 |
| rs35570272 | G | T | 0.604956 | 0.903966 | 0.011885 | 2E-17 | -0.0125 | 0.023742 | 0.5985 |
| rs3785356 | C | T | 0.703372 | 0.896086 | 0.012727 | 6.6E-18 | -0.0222 | 0.025353 | 0.3812 |
| rs3856439 | C | T | 0.656887 | 1.11341 | 0.012209 | 1.4E-18 | 0.0005 | 0.027144 | 0.9853 |
| rs42403 | C | T | 0.887006 | 0.869917 | 0.018294 | 2.6E-14 | 0.086397 | 0.036838 | 0.01901 |
| rs4319131 | A | G | 0.544753 | 1.07054 | 0.011651 | 4.9E-09 | 0.038201 | 0.023045 | 0.09739 |
| rs45613035 | T | C | 0.903289 | 0.833266 | 0.019553 | 1.1E-20 | -0.0861 | 0.042072 | 0.0407 |
| rs4574025 | C | T | 0.465373 | 0.915721 | 0.011658 | 4.3E-14 | -0.0084 | 0.023792 | 0.7239 |
| rs4722758 | C | G | 0.800574 | 0.887831 | 0.014435 | 1.7E-16 | -0.0598 | 0.027334 | 0.02868 |
| rs4792846 | G | A | 0.459444 | 1.08451 | 0.011645 | 3.2E-12 | 0.0395 | 0.024219 | 0.1029 |
| rs4795399 | T | C | 0.530853 | 1.30283 | 0.011583 | 1.9E-115 | -0.1297 | 0.022737 | 1.17E-08 |
| rs479844 | A | G | 0.446616 | 0.895698 | 0.011623 | 2.6E-21 | 0.034305 | 0.022946 | 0.1349 |
| rs4916534 | A | G | 0.91899 | 1.12664 | 0.021354 | 2.3E-08 | 0.023101 | 0.049705 | 0.6421 |
| rs4917131 | A | G | 0.190569 | 1.08637 | 0.014876 | 2.6E-08 | -0.1022 | 0.030555 | 0.000824 |
| rs55743914 | C | T | 0.757827 | 0.904782 | 0.013575 | 1.7E-13 | 0.038595 | 0.02758 | 0.1617 |
| rs56328339 | C | T | 0.836301 | 1.10129 | 0.015672 | 7.5E-10 | 0.1455 | 0.033444 | 1.36E-05 |
| rs62408233 | G | A | 0.643957 | 1.11297 | 0.01205 | 6.5E-19 | 0.110005 | 0.024194 | 5.45E-06 |
| rs6846348 | G | A | 0.686305 | 1.08507 | 0.012659 | 1.1E-10 | 0.042 | 0.02426 | 0.08341 |
| rs6927172 | C | G | 0.779472 | 1.08976 | 0.013934 | 6.9E-10 | -0.0389 | 0.028028 | 0.1652 |
| rs6954667 | C | A | 0.692969 | 0.931182 | 0.012569 | 1.4E-08 | -0.0025 | 0.025533 | 0.9221 |
| rs705700 | T | C | 0.57995 | 0.90236 | 0.011723 | 1.9E-18 | 0.016405 | 0.023348 | 0.4823 |
| rs7209400 | C | T | 0.455041 | 0.924102 | 0.011658 | 1.3E-11 | -0.0109 | 0.022922 | 0.6344 |
| rs72743461 | C | A | 0.767112 | 0.836304 | 0.013702 | 6.7E-39 | -0.1909 | 0.026991 | 1.52E-12 |
| rs72823641 | T | A | 0.857876 | 1.34076 | 0.016524 | 1.8E-70 | 0.035803 | 0.032587 | 0.2719 |
| rs73192661 | C | T | 0.549501 | 0.881349 | 0.011646 | 2.1E-27 | 0.045897 | 0.023185 | 0.04775 |
| rs7582757 | A | G | 0.524664 | 0.937076 | 0.011573 | 2E-08 | -0.0433 | 0.022915 | 0.05879 |
| rs7625643 | A | G | 0.556922 | 0.934908 | 0.011791 | 1.1E-08 | -0.09291 | 0.023474 | 7.56E-05 |
| rs7626218 | A | T | 0.602573 | 1.08332 | 0.011823 | 1.3E-11 | -0.0093 | 0.023261 | 0.6892 |
| rs7848215 | C | T | 0.740551 | 0.796999 | 0.013274 | 1.7E-65 | -0.0362 | 0.025755 | 0.1599 |
| rs7975763 | C | T | 0.793332 | 1.08807 | 0.014324 | 3.8E-09 | -0.0414 | 0.027742 | 0.1356 |
| rs835673 | T | C | 0.265721 | 0.931038 | 0.013093 | 4.8E-08 | 0.076804 | 0.025574 | 0.002672 |
| rs912425 | G | A | 0.239061 | 1.08376 | 0.01359 | 3.2E-09 | 0.103296 | 0.02598 | 7.01E-05 |
| rs9372120 | T | G | 0.7934 | 0.904475 | 0.014294 | 2.2E-12 | -0.0108 | 0.029186 | 0.7114 |
| rs9391997 | A | G | 0.469864 | 0.928512 | 0.011564 | 1.4E-10 | -0.0325 | 0.023204 | 0.1613 |
| rs9662290 | T | C | 0.672699 | 0.932691 | 0.012669 | 3.8E-08 | 0.004599 | 0.026172 | 0.8605 |
| **childhood-onset asthma on UC** | | | | | | | | | |
| rs10118244 | C | T | 0.971414 | 1.21258 | 0.034716 | 2.8E-08 | 0.060196 | 0.062065 | 0.3321 |
| rs10158467 | A | G | 0.714141 | 0.896019 | 0.012885 | 1.6E-17 | -0.0304 | 0.023551 | 0.1968 |
| rs10786050 | A | G | 0.406743 | 0.917752 | 0.011754 | 2.8E-13 | -0.0501 | 0.021557 | 0.02011 |
| rs10836538 | G | T | 0.651655 | 1.08321 | 0.012173 | 5.2E-11 | 0.030202 | 0.022236 | 0.1744 |
| rs10995245 | G | A | 0.652823 | 0.931956 | 0.012168 | 7E-09 | 0.016699 | 0.022242 | 0.4528 |
| rs11065979 | C | T | 0.558799 | 1.08502 | 0.011655 | 2.5E-12 | -0.0639 | 0.02183 | 0.003419 |
| rs11071559 | C | T | 0.868457 | 1.18496 | 0.017078 | 2.9E-23 | 0.092302 | 0.031722 | 0.003618 |
| rs11135015 | T | C | 0.670957 | 0.934697 | 0.012296 | 4E-08 | -0.0841 | 0.023334 | 0.000313 |
| rs11236797 | C | A | 0.552697 | 0.823406 | 0.011634 | 1.3E-62 | -0.1382 | 0.021391 | 1.04E-10 |
| rs11593589 | C | A | 0.457859 | 1.07221 | 0.011592 | 1.8E-09 | -0.0997 | 0.021736 | 4.5E-06 |
| rs11658582 | C | G | 0.379145 | 0.912738 | 0.011907 | 1.7E-14 | -0.065 | 0.021824 | 0.002899 |
| rs12023876 | G | T | 0.667519 | 1.07987 | 0.01226 | 3.7E-10 | -0.0317 | 0.0226 | 0.1607 |
| rs12365699 | G | A | 0.831274 | 1.14126 | 0.015499 | 1.5E-17 | 0.007599 | 0.029072 | 0.7938 |
| rs12531500 | A | G | 0.565634 | 1.10309 | 0.011689 | 4.7E-17 | -0.0219 | 0.021419 | 0.3066 |
| rs12657787 | G | A | 0.847423 | 0.849237 | 0.01615 | 4.6E-24 | -0.0697 | 0.028897 | 0.01586 |
| rs12935657 | G | A | 0.749187 | 1.15436 | 0.013371 | 6.9E-27 | 0.021295 | 0.024155 | 0.378 |
| rs13277355 | A | G | 0.274331 | 1.10297 | 0.012996 | 4.7E-14 | -0.0659 | 0.024678 | 0.007571 |
| rs1612986 | T | C | 0.816653 | 0.856328 | 0.015037 | 6E-25 | -0.0167 | 0.027269 | 0.5403 |
| rs1617333 | A | G | 0.60217 | 1.09812 | 0.011863 | 3E-15 | 0.0278 | 0.023135 | 0.2295 |
| rs1655558 | T | G | 0.454064 | 0.919987 | 0.011706 | 1E-12 | 0.070896 | 0.0221 | 0.001337 |
| rs16903574 | C | G | 0.924845 | 0.839851 | 0.022396 | 6.5E-15 | 0.006598 | 0.041641 | 0.8741 |
| rs17103286 | A | G | 0.478517 | 0.926643 | 0.011603 | 5.2E-11 | -0.0448 | 0.021087 | 0.03363 |
| rs1806656 | C | G | 0.681098 | 1.08202 | 0.012509 | 2.9E-10 | 0.018095 | 0.023765 | 0.4464 |
| rs1837253 | T | C | 0.262925 | 0.846361 | 0.013128 | 5.5E-37 | -0.03319 | 0.023851 | 0.164 |
| rs1885013 | G | A | 0.281984 | 1.09787 | 0.012849 | 3.7E-13 | -0.027 | 0.023133 | 0.2431 |
| rs1887704 | C | G | 0.32215 | 0.907394 | 0.012388 | 4.3E-15 | -0.0006 | 0.021864 | 0.9781 |
| rs1893380 | G | C | 0.6208 | 0.92337 | 0.011913 | 2.2E-11 | -0.0634 | 0.021678 | 0.00345 |
| rs1962588 | T | G | 0.791595 | 0.922422 | 0.014396 | 2E-08 | 0.005505 | 0.027457 | 0.8411 |
| rs2052690 | G | T | 0.712557 | 0.928894 | 0.012785 | 8E-09 | -0.0324 | 0.023191 | 0.1624 |
| rs2066844 | C | T | 0.953476 | 0.853589 | 0.027433 | 7.9E-09 | 0.056697 | 0.052232 | 0.2777 |
| rs2070901 | G | T | 0.731764 | 0.914937 | 0.013062 | 1E-11 | 0.0003 | 0.020637 | 0.9884 |
| rs2202750 | T | C | 0.381027 | 1.11173 | 0.011896 | 5.4E-19 | -0.0644 | 0.021697 | 0.002996 |
| rs2299012 | A | C | 0.808317 | 0.839763 | 0.014686 | 1.3E-32 | -0.0069 | 0.026308 | 0.793 |
| rs230495 | A | G | 0.412214 | 0.936226 | 0.011749 | 2E-08 | 0.075803 | 0.021388 | 0.000394 |
| rs2738783 | T | G | 0.212924 | 0.919593 | 0.014227 | 3.8E-09 | -0.1802 | 0.030616 | 3.96E-09 |
| rs274943 | T | C | 0.516893 | 1.07957 | 0.01159 | 4E-11 | -0.0079 | 0.021012 | 0.7069 |
| rs2765414 | T | G | 0.225532 | 1.09099 | 0.013821 | 2.9E-10 | 0.026301 | 0.024882 | 0.2905 |
| rs2766667 | T | C | 0.256063 | 1.07946 | 0.013289 | 8.7E-09 | -0.0186 | 0.024361 | 0.4451 |
| rs2918302 | G | A | 0.846221 | 0.903596 | 0.016049 | 2.7E-10 | -0.065 | 0.028839 | 0.0242 |
| rs2955118 | G | A | 0.725936 | 1.08175 | 0.013029 | 1.6E-09 | -0.0268 | 0.023493 | 0.254 |
| rs3122929 | C | T | 0.600485 | 0.876303 | 0.011843 | 7.2E-29 | -0.0017 | 0.021837 | 0.938 |
| rs340931 | G | A | 0.327612 | 0.905216 | 0.012327 | 6.6E-16 | 0.003697 | 0.022277 | 0.8682 |
| rs35570272 | G | T | 0.604956 | 0.903966 | 0.011885 | 2E-17 | -0.0064 | 0.02182 | 0.7693 |
| rs3785356 | C | T | 0.703372 | 0.896086 | 0.012727 | 6.6E-18 | -0.0007 | 0.024378 | 0.9771 |
| rs3856439 | C | T | 0.656887 | 1.11341 | 0.012209 | 1.4E-18 | -0.0423 | 0.022522 | 0.06034 |
| rs42403 | C | T | 0.887006 | 0.869917 | 0.018294 | 2.6E-14 | 0.044401 | 0.033246 | 0.1817 |
| rs4319131 | A | G | 0.544753 | 1.07054 | 0.011651 | 4.9E-09 | 0.010396 | 0.021287 | 0.6253 |
| rs45613035 | T | C | 0.903289 | 0.833266 | 0.019553 | 1.1E-20 | -0.0791 | 0.038817 | 0.04158 |
| rs4574025 | C | T | 0.465373 | 0.915721 | 0.011658 | 4.3E-14 | 0.047805 | 0.021674 | 0.02741 |
| rs4722758 | C | G | 0.800574 | 0.887831 | 0.014435 | 1.7E-16 | 0.014997 | 0.02581 | 0.5612 |
| rs4792846 | G | A | 0.459444 | 1.08451 | 0.011645 | 3.2E-12 | 0.021602 | 0.022121 | 0.3288 |
| rs4795399 | T | C | 0.530853 | 1.30283 | 0.011583 | 1.9E-115 | -0.1383 | 0.021149 | 6.19E-11 |
| rs479844 | A | G | 0.446616 | 0.895698 | 0.011623 | 2.6E-21 | 0.0004 | 0.018992 | 0.9832 |
| rs4916534 | A | G | 0.91899 | 1.12664 | 0.021354 | 2.3E-08 | -0.044 | 0.042221 | 0.2973 |
| rs4917131 | A | G | 0.190569 | 1.08637 | 0.014876 | 2.6E-08 | -0.0813 | 0.028125 | 0.003846 |
| rs55743914 | C | T | 0.757827 | 0.904782 | 0.013575 | 1.7E-13 | -0.0017 | 0.026094 | 0.9481 |
| rs56328339 | C | T | 0.836301 | 1.10129 | 0.015672 | 7.5E-10 | 0.055999 | 0.030308 | 0.06465 |
| rs62408233 | G | A | 0.643957 | 1.11297 | 0.01205 | 6.5E-19 | 0.070701 | 0.022317 | 0.001535 |
| rs6846348 | G | A | 0.686305 | 1.08507 | 0.012659 | 1.1E-10 | 0.018602 | 0.022602 | 0.4105 |
| rs6927172 | C | G | 0.779472 | 1.08976 | 0.013934 | 6.9E-10 | -0.165 | 0.025074 | 4.68E-11 |
| rs6954667 | C | A | 0.692969 | 0.931182 | 0.012569 | 1.4E-08 | 0.023996 | 0.02365 | 0.3103 |
| rs705700 | T | C | 0.57995 | 0.90236 | 0.011723 | 1.9E-18 | 0.007403 | 0.021449 | 0.73 |
| rs7209400 | C | T | 0.455041 | 0.924102 | 0.011658 | 1.3E-11 | 0.019499 | 0.021156 | 0.3567 |
| rs72743461 | C | A | 0.767112 | 0.836304 | 0.013702 | 6.7E-39 | -0.106 | 0.024841 | 1.98E-05 |
| rs72823641 | T | A | 0.857876 | 1.34076 | 0.016524 | 1.8E-70 | 0.009596 | 0.030291 | 0.7514 |
| rs73192661 | C | T | 0.549501 | 0.881349 | 0.011646 | 2.1E-27 | -0.0151 | 0.021353 | 0.4796 |
| rs7582757 | A | G | 0.524664 | 0.937076 | 0.011573 | 2E-08 | -0.034 | 0.021197 | 0.1087 |
| rs7625643 | A | G | 0.556922 | 0.934908 | 0.011791 | 1.1E-08 | 0.015804 | 0.021816 | 0.4688 |
| rs7626218 | A | T | 0.602573 | 1.08332 | 0.011823 | 1.3E-11 | -0.0058 | 0.02135 | 0.786 |
| rs7848215 | C | T | 0.740551 | 0.796999 | 0.013274 | 1.7E-65 | -0.0083 | 0.024167 | 0.7314 |
| rs7975763 | C | T | 0.793332 | 1.08807 | 0.014324 | 3.8E-09 | -0.0034 | 0.025967 | 0.8957 |
| rs835673 | T | C | 0.265721 | 0.931038 | 0.013093 | 4.8E-08 | 0.038798 | 0.023878 | 0.1042 |
| rs912425 | G | A | 0.239061 | 1.08376 | 0.01359 | 3.2E-09 | -0.0112 | 0.024752 | 0.651 |
| rs9372120 | T | G | 0.7934 | 0.904475 | 0.014294 | 2.2E-12 | 0.038596 | 0.026686 | 0.1481 |
| rs9391997 | A | G | 0.469864 | 0.928512 | 0.011564 | 1.4E-10 | 0.008801 | 0.021366 | 0.6804 |
| rs9662290 | T | C | 0.672699 | 0.932691 | 0.012669 | 3.8E-08 | -0.0345 | 0.023588 | 0.1436 |

**Table S4** Genetic variants used in the analyses investigating a causal impact of genetically predicted adult-onset asthma and inflammatory bowel disease (IBD) including its subgroups Crohn´s disease (CD) and ulcerative colitis (UC)

| **SNP** | **EA** | **OA** | **EAF** | $\boldsymbol{\beta}$**_expsr_** | **SE_expsr_** | $\boldsymbol{P}$**_expsr_** | $\boldsymbol{\beta}$**_otcm_** | **SE_otcm_** | $\boldsymbol{P}$**_otcm_** |
| --- | --- | --- | --- | --- | --- | --- | --- | --- | --- |
| **adult-onset asthma on IBD** | | | | | | | | | |
| rs1041973 | C | A | 0.770787 | 1.06917 | 0.009862 | 1.2E-11 | 0.026498 | 0.0196 | 0.1764 |
| rs1059513 | T | C | 0.891157 | 1.11949 | 0.013352 | 2.8E-17 | 0.020303 | 0.02819 | 0.4714 |
| rs10795672 | G | A | 0.5304 | 0.942709 | 0.008483 | 3.5E-12 | -0.0145 | 0.018551 | 0.4343 |
| rs11088309 | C | G | 0.858141 | 0.919155 | 0.011921 | 1.5E-12 | -0.0172 | 0.024376 | 0.4805 |
| rs11168252 | T | A | 0.759795 | 1.05727 | 0.009774 | 1.2E-08 | -0.0501 | 0.020439 | 0.01423 |
| rs11715524 | G | A | 0.490248 | 0.949974 | 0.008366 | 8.5E-10 | -0.0163 | 0.018072 | 0.3672 |
| rs11742240 | G | T | 0.722258 | 1.06323 | 0.009291 | 4.1E-11 | 0.020805 | 0.019079 | 0.2755 |
| rs12470864 | G | A | 0.616213 | 0.905753 | 0.008569 | 7.2E-31 | 0.092302 | 0.017663 | 1.73E-07 |
| rs13241235 | T | C | 0.642443 | 1.05041 | 0.008679 | 1.5E-08 | -0.0127 | 0.017555 | 0.4694 |
| rs174535 | T | C | 0.648949 | 1.06561 | 0.008722 | 3.2E-13 | -0.0696 | 0.017665 | 8.15E-05 |
| rs17454584 | A | G | 0.7862 | 0.937118 | 0.010138 | 1.5E-10 | -0.0947 | 0.01997 | 2.12E-06 |
| rs17668708 | C | T | 0.892707 | 1.09563 | 0.013551 | 1.6E-11 | -0.0466 | 0.027049 | 0.08494 |
| rs1784775 | T | C | 0.291663 | 0.946886 | 0.009155 | 2.5E-09 | 0.005505 | 0.018255 | 0.763 |
| rs1898671 | C | T | 0.652732 | 0.924602 | 0.008728 | 2.7E-19 | -0.0161 | 0.017925 | 0.3691 |
| rs2056625 | G | A | 0.589775 | 1.06049 | 0.008449 | 3.6E-12 | 0.002102 | 0.016814 | 0.9005 |
| rs2338821 | A | G | 0.384603 | 0.95189 | 0.008555 | 8.2E-09 | -0.0743 | 0.017444 | 2.05E-05 |
| rs2381712 | T | G | 0.479898 | 0.947059 | 0.008327 | 6.5E-11 | -0.0384 | 0.017898 | 0.03192 |
| rs28635831 | A | G | 0.649888 | 1.05109 | 0.008761 | 1.3E-08 | 0.071502 | 0.017833 | 6.09E-05 |
| rs301816 | G | A | 0.417313 | 0.953193 | 0.008475 | 1.5E-08 | 0.0009 | 0.017601 | 0.9592 |
| rs3024655 | A | G | 0.061738 | 0.882737 | 0.017371 | 7E-13 | 0.0446 | 0.035884 | 0.2139 |
| rs35441874 | T | A | 0.747667 | 1.08093 | 0.00962 | 6E-16 | 0.013004 | 0.019106 | 0.4961 |
| rs4099209 | T | G | 0.846519 | 0.917777 | 0.011555 | 1.1E-13 | -0.039 | 0.021907 | 0.07503 |
| rs4491851 | G | A | 0.466068 | 0.946879 | 0.008371 | 7E-11 | -0.0145 | 0.017013 | 0.3939 |
| rs4771332 | T | C | 0.316872 | 0.942927 | 0.009001 | 6.6E-11 | 0.017496 | 0.018451 | 0.343 |
| rs4947324 | C | T | 0.897819 | 1.08766 | 0.013707 | 8.8E-10 | 0.043095 | 0.027883 | 0.1222 |
| rs57585717 | G | A | 0.881048 | 0.926149 | 0.012843 | 2.3E-09 | 0.003596 | 0.025152 | 0.8863 |
| rs62296577 | C | T | 0.668518 | 1.05321 | 0.008849 | 4.7E-09 | -0.0034 | 0.017413 | 0.845 |
| rs66632892 | G | A | 0.740959 | 0.946701 | 0.009554 | 9.9E-09 | -0.0147 | 0.019565 | 0.4524 |
| rs6866614 | A | G | 0.426146 | 0.929859 | 0.008487 | 1E-17 | 0.136696 | 0.017023 | 9.75E-16 |
| rs7183955 | A | C | 0.811033 | 1.06955 | 0.010676 | 3E-10 | 0.047704 | 0.021877 | 0.02922 |
| rs72743461 | C | A | 0.76717 | 0.911835 | 0.009861 | 8E-21 | -0.1491 | 0.019868 | 6.17E-14 |
| rs7302200 | G | A | 0.662158 | 0.934094 | 0.008813 | 1E-14 | -0.0028 | 0.018125 | 0.8774 |
| rs7824278 | C | A | 0.387277 | 1.06233 | 0.008539 | 1.4E-12 | -0.0446 | 0.017156 | 0.009332 |
| rs7936312 | G | T | 0.527401 | 0.918629 | 0.008337 | 2.4E-24 | -0.1545 | 0.017071 | 1.43E-19 |
| rs891058 | G | A | 0.70397 | 1.05126 | 0.00912 | 4.2E-08 | -0.0288 | 0.018679 | 0.1231 |
| rs943689 | C | T | 0.644135 | 1.07349 | 0.00867 | 2.8E-16 | 0.086997 | 0.017724 | 9.18E-07 |
| rs992969 | A | G | 0.250135 | 1.11548 | 0.00963 | 7.6E-30 | 0.025804 | 0.019096 | 0.1766 |
| **adult-onset asthma on CD** | | | | | | | | | |
| rs1041973 | C | A | 0.770787 | 1.06917 | 0.009862 | 1.2E-11 | 0.048896 | 0.026349 | 0.06349 |
| rs1059513 | T | C | 0.891157 | 1.11949 | 0.013352 | 2.8E-17 | 0.014504 | 0.038991 | 0.7099 |
| rs10795672 | G | A | 0.5304 | 0.942709 | 0.008483 | 3.5E-12 | -0.023 | 0.025162 | 0.3606 |
| rs11088309 | C | G | 0.858141 | 0.919155 | 0.011921 | 1.5E-12 | -0.0407 | 0.032593 | 0.2118 |
| rs11168252 | T | A | 0.759795 | 1.05727 | 0.009774 | 1.2E-08 | -0.0317 | 0.028334 | 0.2632 |
| rs11715524 | G | A | 0.490248 | 0.949974 | 0.008366 | 8.5E-10 | 0.025502 | 0.025042 | 0.3085 |
| rs11742240 | G | T | 0.722258 | 1.06323 | 0.009291 | 4.1E-11 | 0.010101 | 0.025914 | 0.6967 |
| rs12470864 | G | A | 0.616213 | 0.905753 | 0.008569 | 7.2E-31 | 0.1207 | 0.024097 | 5.47E-07 |
| rs13241235 | T | C | 0.642443 | 1.05041 | 0.008679 | 1.5E-08 | 0.009703 | 0.024129 | 0.6876 |
| rs174535 | T | C | 0.648949 | 1.06561 | 0.008722 | 3.2E-13 | -0.1182 | 0.023933 | 7.86E-07 |
| rs17454584 | A | G | 0.7862 | 0.937118 | 0.010138 | 1.5E-10 | -0.1003 | 0.027316 | 0.000241 |
| rs17668708 | C | T | 0.892707 | 1.09563 | 0.013551 | 1.6E-11 | -0.1016 | 0.03614 | 0.004935 |
| rs1775554 | A | C | 0.573108 | 1.10914 | 0.008413 | 7.8E-35 | 0.0003 | 0.020281 | 0.9882 |
| rs1784775 | T | C | 0.291663 | 0.946886 | 0.009155 | 2.5E-09 | -0.0183 | 0.024974 | 0.4638 |
| rs1898671 | C | T | 0.652732 | 0.924602 | 0.008728 | 2.7E-19 | -0.0053 | 0.024433 | 0.8284 |
| rs2056625 | G | A | 0.589775 | 1.06049 | 0.008449 | 3.6E-12 | -0.036 | 0.023307 | 0.1224 |
| rs2338821 | A | G | 0.384603 | 0.95189 | 0.008555 | 8.2E-09 | -0.1262 | 0.023937 | 1.35E-07 |
| rs2381712 | T | G | 0.479898 | 0.947059 | 0.008327 | 6.5E-11 | 0.002098 | 0.023949 | 0.9302 |
| rs28635831 | A | G | 0.649888 | 1.05109 | 0.008761 | 1.3E-08 | 0.047704 | 0.024368 | 0.05027 |
| rs301816 | G | A | 0.417313 | 0.953193 | 0.008475 | 1.5E-08 | -0.0571 | 0.023444 | 0.01487 |
| rs3024655 | A | G | 0.061738 | 0.882737 | 0.017371 | 7E-13 | 0.045098 | 0.049165 | 0.359 |
| rs35441874 | T | A | 0.747667 | 1.08093 | 0.00962 | 6E-16 | -0.0253 | 0.025644 | 0.3239 |
| rs4099209 | T | G | 0.846519 | 0.917777 | 0.011555 | 1.1E-13 | 0.011296 | 0.029627 | 0.703 |
| rs4491851 | G | A | 0.466068 | 0.946879 | 0.008371 | 7E-11 | 0.018796 | 0.023672 | 0.4272 |
| rs4771332 | T | C | 0.316872 | 0.942927 | 0.009001 | 6.6E-11 | 0.014504 | 0.024873 | 0.5598 |
| rs4947324 | C | T | 0.897819 | 1.08766 | 0.013707 | 8.8E-10 | -0.09 | 0.036462 | 0.01358 |
| rs57585717 | G | A | 0.881048 | 0.926149 | 0.012843 | 2.3E-09 | -0.0011 | 0.033994 | 0.9742 |
| rs62296577 | C | T | 0.668518 | 1.05321 | 0.008849 | 4.7E-09 | -0.0005 | 0.023187 | 0.9828 |
| rs66632892 | G | A | 0.740959 | 0.946701 | 0.009554 | 9.9E-09 | -0.0113 | 0.027002 | 0.6757 |
| rs6866614 | A | G | 0.426146 | 0.929859 | 0.008487 | 1E-17 | 0.2126 | 0.023052 | 2.9E-20 |
| rs7183955 | A | C | 0.811033 | 1.06955 | 0.010676 | 3E-10 | 0.017997 | 0.02989 | 0.5471 |
| rs72743461 | C | A | 0.76717 | 0.911835 | 0.009861 | 8E-21 | -0.1909 | 0.026991 | 1.52E-12 |
| rs7302200 | G | A | 0.662158 | 0.934094 | 0.008813 | 1E-14 | -0.0045 | 0.024955 | 0.8569 |
| rs7824278 | C | A | 0.387277 | 1.06233 | 0.008539 | 1.4E-12 | -0.0185 | 0.023127 | 0.4238 |
| rs7936312 | G | T | 0.527401 | 0.918629 | 0.008337 | 2.4E-24 | -0.1773 | 0.023303 | 2.78E-14 |
| rs891058 | G | A | 0.70397 | 1.05126 | 0.00912 | 4.2E-08 | -0.0014 | 0.025947 | 0.957 |
| rs943689 | C | T | 0.644135 | 1.07349 | 0.00867 | 2.8E-16 | 0.110005 | 0.024269 | 5.83E-06 |
| rs992969 | A | G | 0.250135 | 1.11548 | 0.00963 | 7.6E-30 | 0.043998 | 0.025886 | 0.08919 |
| **adult-onset asthma on UC** | | | | | | | | | |
| rs1041973 | C | A | 0.770787 | 1.06917 | 0.009862 | 1.2E-11 | 0.014697 | 0.024792 | 0.5533 |
| rs1059513 | T | C | 0.891157 | 1.11949 | 0.013352 | 2.8E-17 | 0.012896 | 0.035292 | 0.7148 |
| rs10795672 | G | A | 0.5304 | 0.942709 | 0.008483 | 3.5E-12 | -0.0022 | 0.023097 | 0.9242 |
| rs11088309 | C | G | 0.858141 | 0.919155 | 0.011921 | 1.5E-12 | -0.0012 | 0.031403 | 0.9695 |
| rs11168252 | T | A | 0.759795 | 1.05727 | 0.009774 | 1.2E-08 | -0.0599 | 0.025544 | 0.01903 |
| rs11715524 | G | A | 0.490248 | 0.949974 | 0.008366 | 8.5E-10 | -0.0536 | 0.022684 | 0.01814 |
| rs11742240 | G | T | 0.722258 | 1.06323 | 0.009291 | 4.1E-11 | 0.023105 | 0.02402 | 0.3361 |
| rs12470864 | G | A | 0.616213 | 0.905753 | 0.008569 | 7.2E-31 | 0.072603 | 0.022243 | 0.001098 |
| rs13241235 | T | C | 0.642443 | 1.05041 | 0.008679 | 1.5E-08 | -0.0298 | 0.022007 | 0.1757 |
| rs174535 | T | C | 0.648949 | 1.06561 | 0.008722 | 3.2E-13 | -0.0378 | 0.022311 | 0.09026 |
| rs17454584 | A | G | 0.7862 | 0.937118 | 0.010138 | 1.5E-10 | -0.09199 | 0.024997 | 0.000233 |
| rs17668708 | C | T | 0.892707 | 1.09563 | 0.013551 | 1.6E-11 | -0.0107 | 0.034341 | 0.7553 |
| rs1775554 | A | C | 0.573108 | 1.10914 | 0.008413 | 7.8E-35 | -0.001 | 0.021626 | 0.9631 |
| rs1784775 | T | C | 0.291663 | 0.946886 | 0.009155 | 2.5E-09 | 0.019303 | 0.023185 | 0.4051 |
| rs1898671 | C | T | 0.652732 | 0.924602 | 0.008728 | 2.7E-19 | -0.0147 | 0.02264 | 0.5161 |
| rs2056625 | G | A | 0.589775 | 1.06049 | 0.008449 | 3.6E-12 | 0.031904 | 0.021667 | 0.1409 |
| rs2338821 | A | G | 0.384603 | 0.95189 | 0.008555 | 8.2E-09 | -0.0484 | 0.021924 | 0.02726 |
| rs2381712 | T | G | 0.479898 | 0.947059 | 0.008327 | 6.5E-11 | -0.069 | 0.022504 | 0.00217 |
| rs28635831 | A | G | 0.649888 | 1.05109 | 0.008761 | 1.3E-08 | 0.082004 | 0.022505 | 0.000269 |
| rs301816 | G | A | 0.417313 | 0.953193 | 0.008475 | 1.5E-08 | 0.0425 | 0.02167 | 0.04985 |
| rs3024655 | A | G | 0.061738 | 0.882737 | 0.017371 | 7E-13 | 0.027197 | 0.045101 | 0.5465 |
| rs35441874 | T | A | 0.747667 | 1.08093 | 0.00962 | 6E-16 | 0.023996 | 0.024159 | 0.3206 |
| rs4099209 | T | G | 0.846519 | 0.917777 | 0.011555 | 1.1E-13 | -0.0677 | 0.027736 | 0.01465 |
| rs4491851 | G | A | 0.466068 | 0.946879 | 0.008371 | 7E-11 | -0.031 | 0.021125 | 0.1422 |
| rs4771332 | T | C | 0.316872 | 0.942927 | 0.009001 | 6.6E-11 | 0.017103 | 0.023319 | 0.4633 |
| rs4947324 | C | T | 0.897819 | 1.08766 | 0.013707 | 8.8E-10 | 0.160497 | 0.036277 | 9.68E-06 |
| rs57585717 | G | A | 0.881048 | 0.926149 | 0.012843 | 2.3E-09 | 0.017095 | 0.03165 | 0.5891 |
| rs62296577 | C | T | 0.668518 | 1.05321 | 0.008849 | 4.7E-09 | -0.0082 | 0.022234 | 0.7124 |
| rs66632892 | G | A | 0.740959 | 0.946701 | 0.009554 | 9.9E-09 | -0.0168 | 0.024577 | 0.4943 |
| rs6866614 | A | G | 0.426146 | 0.929859 | 0.008487 | 1E-17 | 0.089603 | 0.021457 | 2.97E-05 |
| rs7183955 | A | C | 0.811033 | 1.06955 | 0.010676 | 3E-10 | 0.0419 | 0.0275 | 0.1276 |
| rs72743461 | C | A | 0.76717 | 0.911835 | 0.009861 | 8E-21 | -0.106 | 0.024841 | 1.98E-05 |
| rs7302200 | G | A | 0.662158 | 0.934094 | 0.008813 | 1E-14 | -0.0045 | 0.023275 | 0.8467 |
| rs7824278 | C | A | 0.387277 | 1.06233 | 0.008539 | 1.4E-12 | -0.065 | 0.021755 | 0.00281 |
| rs7936312 | G | T | 0.527401 | 0.918629 | 0.008337 | 2.4E-24 | -0.1386 | 0.021457 | 1.05E-10 |
| rs891058 | G | A | 0.70397 | 1.05126 | 0.00912 | 4.2E-08 | -0.0368 | 0.023428 | 0.1162 |
| rs943689 | C | T | 0.644135 | 1.07349 | 0.00867 | 2.8E-16 | 0.070498 | 0.022352 | 0.001611 |
| rs992969 | A | G | 0.250135 | 1.11548 | 0.00963 | 7.6E-30 | 0.007204 | 0.024281 | 0.7667 |

**Table S5** Identified pleiotropic SNPs of the association between genetically predicted childhood-onset asthma and gastrointestinal disorders based on the significance threshold $\alpha$ = 0.01.

| **Iteration** | **Pleiotropic SNP** | **Q statistic** | ***P*-value** | **Confounder-association** |
| --- | --- | --- | --- | --- |
| **childhood-onset asthma on IBS** | | | | |
| 1 | rs12531500 | 7.471 | 6.27E-03 | allergic diseases, bronchitis |
| 1 | rs2765414 | 8.318 | 3.92E-03 | allergic diseases |
| 1 | rs7975763 | 17.294 | 3.20E-05 | allergic diseases, bronchitis, blood pressure, schizophrenia, fat-free mass, education |
| 1 | rs892225 | 7.739 | 5.40E-03 | reticulocyte fraction of red cells, heel bone mineral density |
| **childhood-onset asthma on IBD** | | | | |
| 1 | rs10187276 | 8.712 | 3.16E-03 | allergic diseases, bronchitis |
| 1 | rs10836538 | 9.085 | 2.58E-03 | allergic diseases, bronchitis |
| 1 | rs11236797 | 32.566 | 1.15E-08 | allergic diseases, bronchitis, treatment with ventolin |
| 1 | rs1806656 | 10.818 | 1.01E-03 | allergic diseases, bronchitis |
| 1 | rs2066844 | 9.377 | 2.20E-03 | Mouth or teeth dental problems: mouth ulcers |
| 1 | rs230495 | 12.671 | 3.71E-04 | lymphocyte/neutrophil percentage of white cells, hayfever, allergic rhinitis or eczema |
| 1 | rs2738783 | 14.641 | 1.30E-04 | allergic diseases, bronchitis |
| 1 | rs35570272 | 9.955 | 1.60E-03 | allergic diseases, bronchitis |
| 1 | rs4319131 | 8.012 | 4.65E-03 | bone mineral density |
| 1 | rs4795399 | 7.889 | 4.97E-03 | allergic diseases, bronchitis, HDL, treatment with ventolin |
| 1 | rs4917131 | 8.000 | 4.68E-03 | granulocyte/monocyte percentage of white cells |
| 1 | rs56328339 | 7.044 | 7.95E-03 | allergic diseases, bronchitis |
| 1 | rs62408233 | 9.061 | 2.61E-03 | allergic diseases, bronchitis |
| 1 | rs6927172 | 29.666 | 5.13E-08 | rheumatoid arthritis, lymphocyte percentage of white cells |
| 1 | rs72743461 | 15.537 | 8.09E-05 | coronary artery disease, allergic diseases, bronchitis, treatment with ventolin |
| 1 | rs835673 | 14.261 | 1.59E-04 | allergic diseases, bronchitis |
| 2 | rs11135015 | 6.637 | 9.99E-03 | hematocrit, red blood cell count, self-reported kidney stone |
| **childhood-onset asthma on PUD** | | | | |
| 1 | rs12365699 | 7.787 | 5.26E-03 | allergic diseases, bronchitis, atopic dermatitis |
| 1 | rs1617333 | 6.645 | 9.95E-03 | allergic diseases, bronchitis |
| 1 | rs2765414 | 7.306 | 6.87E-03 | allergic diseases |
| **childhood-onset asthma on GORD** | | | | |
| 1 | rs11065979 | 8.546 | 3.46E-03 | medication use |
| 1 | rs12123821 | 7.287 | 6.94E-03 | allergic diseases, bronchitis |
| 1 | rs2738783 | 8.217 | 4.15E-03 | allergic diseases, bronchitis |
| 1 | rs34290285 | 7.401 | 6.52E-03 | allergic diseases, bronchitis, no blood clot |
| 1 | rs705700 | 15.130 | 1.00E-04 | allergic diseases, bronchitis, fat-free mass, no blood clot |
| 1 | rs892225 | 7.493 | 6.19E-03 | reticulocyte fraction of red cells, heel bone mineral density |

**Table S6** Identified pleiotropic SNPs of the association between genetically predicted adult-onset asthma and gastrointestinal disorders based on the significance threshold $\alpha$ = 0.01.

| **Iteration** | **Pleiotropic SNP** | **Q statistic** | ***P*-value** | **Confounder-association** |
| --- | --- | --- | --- | --- |
| **adult-onset asthma on IBS** | | | | |
| 1 | rs301816 | 11.894 | 5.63E-04 | allergic diseases, bronchitis, no blood clot |
| **adult-onset asthma on IBD** | | | | |
| 1 | rs12470864 | 9.282 | 2.31E-03 | allergic diseases, bronchitis, treatment with ventolin, no blood clot |
| 1 | rs17454584 | 10.479 | 1.21E-03 | allergic diseases, bronchitis, no blood clot |
| 1 | rs4947324 | 8.225 | 4.13E-03 | allergic diseases, bronchitis, no blood clot |
| 1 | rs6866614 | 24.543 | 7.27E-07 | allergic diseases, bronchitis, fat-free mass |
| 1 | rs72743461 | 17.302 | 3.19E-05 | coronary artery disease, allergic diseases, bronchitis, treatment with ventolin |
| 1 | rs7824278 | 9.386 | 2.19E-03 | allergic diseases, bronchitis, no blood clot |
| 1 | rs7936312 | 39.962 | 2.59E-10 | allergic diseases, bronchitis, no blood clot |
| 1 | rs8067124 | 7.259 | 7.06E-03 | allergic diseases, bronchitis, no blood clot |
| 1 | rs943689 | 7.192 | 7.32E-03 | allergic diseases, bronchitis, no blood clot |
| 2 | rs8067124 | 7.379 | 6.60E-03 | allergic diseases, bronchitis, no blood clot |
| **adult-onset asthma on PUD** | | | | |
| 1 | rs1784775 | 7.575 | 5.92E-03 | allergic diseases, bronchitis, no blood clot |
| **adult-onset asthma on GORD** | | | | |
| 1 | rs11168252 | 7.533 | 6.06E-03 | hypertension, high blood pressure, allergic diseases |
| 1 | rs34290285 | 8.027 | 4.61E-03 | allergic diseases, bronchitis, no blood clot |
| 1 | rs4491851 | 8.543 | 3.47E-03 | allergic diseases, bronchitis, no blood clot |
| 1 | rs4947324 | 15.607 | 7.80E-05 | allergic diseases, bronchitis, no blood clot |
| 1 | rs62296577 | 8.739 | 3.12E-03 | allergic diseases, fat-free mass |
| 1 | rs7302200 | 9.225 | 2.39E-03 | allergic diseases, bronchitis, no blood clot, fat-free mass |

**Table S7** Identified pleiotropic SNPs of the association between genetically predicted childhood-onset asthma and inflammatory bowel disease (IBD) and its subgroups Crohn´s disease (CD) and ulcerative colitis (UC) based on the significance threshold $\alpha$ = 0.01.

| **Iteration** | **Pleiotropic SNP** | **Q statistic** | ***P*-value** | **Confounder-association** |
| --- | --- | --- | --- | --- |
| **childhood-onset asthma on IBD** | | | | |
| 1 | rs10836538 | 6.833 | 8.95E-03 | allergic diseases, bronchitis |
| 1 | rs11065979 | 8.291 | 3.98E-03 | medication use |
| 1 | rs11071559 | 21.753 | 3.10E-06 | allergic diseases, bronchitis, no blood clot |
| 1 | rs11135015 | 7.874 | 5.02E-03 | hematocrit, red blood cell count, self-reported kidney stone |
| 1 | rs11236797 | 71.109 | 3.38E-17 | allergic diseases, bronchitis, treatment with ventolin |
| 1 | rs11593589 | 14.065 | 1.77E-04 | allergic diseases, bronchitis, no blood clot, waist circumference |
| 1 | rs1655558 | 15.447 | 8.49E-05 | allergic diseases, bronchitis, no blood clot |
| 1 | rs2066844 | 63.609 | 1.52E-15 | Mouth or teeth dental problems: mouth ulcers |
| 1 | rs2738783 | 54.572 | 1.50E-13 | allergic diseases, bronchitis |
| 1 | rs42403 | 8.784 | 3.04E-03 | allergic diseases, bronchitis, no blood clot |
| 1 | rs4795399 | 49.256 | 2.25E-12 | allergic diseases, bronchitis, HDL, treatment with ventolin |
| 1 | rs4917131 | 12.616 | 3.82E-04 | granulocyte/monocyte percentage of white cells |
| 1 | rs56328339 | 16.834 | 4.08E-05 | allergic diseases, bronchitis |
| 1 | rs62408233 | 34.880 | 3.51E-09 | allergic diseases, bronchitis |
| 1 | rs6846348 | 6.986 | 8.22E-03 | allergic diseases, bronchitis, no blood clot |
| 1 | rs6927172 | 21.568 | 3.42E-06 | rheumatoid arthritis, lymphocyte percentage of white cells |
| 1 | rs72743461 | 47.126 | 6.66E-12 | coronary artery disease, allergic diseases, bronchitis, treatment with ventolin |
| 1 | rs835673 | 11.375 | 7.44E-04 | allergic diseases, bronchitis |
| 1 | rs912425 | 9.688 | 1.85E-03 | hypothyroidism or myxoedema, rheumatoid arthritis |
| 2 | rs45613035 | 7.156 | 7.47E-03 | allergic diseases, bronchitis, treatment with ventolin |
| **childhood-onset asthma on CD** | | | | |
| 1 | rs11236797 | 54.298 | 1.72E-13 | allergic diseases, bronchitis, treatment with ventolin |
| 1 | rs2052690 | 7.449 | 6.35E-03 | blood pressure, hemoglobin concentration, medication use |
| 1 | rs2066844 | 191.810 | 1.28E-43 | Mouth or teeth dental problems: mouth ulcers |
| 1 | rs2738783 | 28.200 | 1.09E-07 | allergic diseases, bronchitis |
| 1 | rs42403 | 7.031 | 8.01E-03 | allergic diseases, bronchitis, no blood clot |
| 1 | rs4795399 | 24.612 | 7.01E-07 | allergic diseases, bronchitis, HDL, treatment with ventolin |
| 1 | rs4917131 | 8.315 | 3.93E-03 | granulocyte/monocyte percentage of white cells |
| 1 | rs56328339 | 22.825 | 1.77E-06 | allergic diseases, bronchitis |
| 1 | rs62408233 | 26.454 | 2.70E-07 | allergic diseases, bronchitis |
| 1 | rs72743461 | 44.498 | 2.55E-11 | coronary artery disease, allergic diseases, bronchitis, treatment with ventolin |
| 1 | rs7625643 | 11.841 | 5.79E-04 | waist/hip circumference, fat mass |
| 1 | rs835673 | 12.078 | 5.10E-04 | allergic diseases, bronchitis |
| 1 | rs912425 | 20.404 | 6.27E-06 | hypothyroidism or myxoedema, rheumatoid arthritis |
| **childhood-onset asthma on UC** | | | | |
| 1 | rs11071559 | 12.323 | 4.47E-04 | allergic diseases, bronchitis, no blood clot |
| 1 | rs11135015 | 8.760 | 3.08E-03 | hematocrit, red blood cell count, self-reported kidney stone |
| 1 | rs11236797 | 34.121 | 5.18E-09 | allergic diseases, bronchitis, treatment with ventolin |
| 1 | rs11593589 | 14.388 | 1.49E-04 | allergic diseases, bronchitis, no blood clot, waist circumference |
| 1 | rs1655558 | 15.034 | 1.06E-04 | allergic diseases, bronchitis, no blood clot |
| 1 | rs230495 | 18.046 | 2.16E-05 | lymphocyte/neutrophil percentage of white cells, hayfever, allergic rhinitis or eczema |
| 1 | rs2738783 | 29.187 | 6.57E-08 | allergic diseases, bronchitis |
| 1 | rs4574025 | 8.324 | 3.91E-03 | allergic diseases, bronchitis, no blood clot |
| 1 | rs4795399 | 30.780 | 2.89E-08 | allergic diseases, bronchitis, HDL, treatment with ventolin |
| 1 | rs62408233 | 15.761 | 7.19E-05 | allergic diseases, bronchitis |
| 1 | rs6927172 | 34.590 | 4.07E-09 | rheumatoid arthritis, lymphocyte percentage of white cells |
| 1 | rs72743461 | 13.879 | 1.95E-04 | coronary artery disease, allergic diseases, bronchitis, treatment with ventolin |

**Table S8** Identified pleiotropic SNPs of the association between genetically predicted adult-onset asthma and inflammatory bowel disease (IBD) and its subgroups Crohn´s disease (CD) and ulcerative colitis (UC) based on the significance threshold $\alpha$ = 0.01.

| **Iteration** | **Pleiotropic SNP** | **Q statistic** | ***P*-value** | **Confounder-association** |
| --- | --- | --- | --- | --- |
| **childhood-onset asthma on IBD** | | | | |
| 1 | rs12470864 | 31.006 | 2.57E-08 | allergic diseases, bronchitis, treatment with ventolin, no blood clot |
| 1 | rs174535 | 12.509 | 4.05E-04 |  |
| 1 | rs17454584 | 19.611 | 9.49E-06 | allergic diseases, bronchitis, no blood clot |
| 1 | rs2338821 | 15.171 | 9.82E-05 | allergic diseases, bronchitis, no blood clot |
| 1 | rs28635831 | 19.387 | 1.07E-05 | eosinophil/neutrophil percentage of granulocytes, rheumatoid arthritis |
| 1 | rs6866614 | 70.473 | 4.67E-17 | allergic diseases, bronchitis, fat-free mass |
| 1 | rs72743461 | 51.810 | 6.11E-13 | coronary artery disease, allergic diseases, bronchitis, treatment with ventolin |
| 1 | rs7936312 | 75.524 | 3.61E-18 | allergic diseases, bronchitis, no blood clot |
| 1 | rs943689 | 28.229 | 1.08E-07 | allergic diseases, bronchitis, no blood clot |
| **childhood-onset asthma on CD** | | | | |
| 1 | rs12470864 | 28.439 | 9.67E-08 | allergic diseases, bronchitis, treatment with ventolin, no blood clot |
| 1 | rs174535 | 20.749 | 5.24E-06 |  |
| 1 | rs17454584 | 11.398 | 7.35E-04 | allergic diseases, bronchitis, no blood clot |
| 1 | rs2338821 | 24.299 | 8.25E-07 | allergic diseases, bronchitis, no blood clot |
| 1 | rs6866614 | 91.587 | 1.07E-21 | allergic diseases, bronchitis, fat-free mass |
| 1 | rs72743461 | 45.991 | 1.19E-11 | coronary artery disease, allergic diseases, bronchitis, treatment with ventolin |
| 1 | rs7936312 | 52.834 | 3.63E-13 | allergic diseases, bronchitis, no blood clot |
| 1 | rs943689 | 24.145 | 8.94E-07 | allergic diseases, bronchitis, no blood clot |
| 2 | rs17668708 | 7.317 | 6.83E-03 | eosinophil/neutrophil percentage of granulocytes |
| **childhood-onset asthma on UC** | | | | |
| 1 | rs12470864 | 12.265 | 4.62E-04 | allergic diseases, bronchitis, treatment with ventolin, no blood clot |
| 1 | rs17454584 | 11.979 | 5.38E-04 | allergic diseases, bronchitis, no blood clot |
| 1 | rs2381712 | 7.952 | 4.80E-03 | smoking |
| 1 | rs28635831 | 15.341 | 8.98E-05 | eosinophil/neutrophil percentage of granulocytes, rheumatoid arthritis |
| 1 | rs4947324 | 21.155 | 4.24E-06 | allergic diseases, bronchitis, no blood clot |
| 1 | rs6866614 | 19.618 | 9.46E-06 | allergic diseases, bronchitis, fat-free mass |
| 1 | rs72743461 | 16.423 | 5.07E-05 | coronary artery disease, allergic diseases, bronchitis, treatment with ventolin |
| 1 | rs7824278 | 7.303 | 6.88E-03 | allergic diseases, bronchitis, no blood clot |
| 1 | rs7936312 | 38.550 | 5.34E-10 | allergic diseases, bronchitis, no blood clot |
| 1 | rs943689 | 11.796 | 5.93E-04 | allergic diseases, bronchitis, no blood clot |

**Table S9** Estimates, standard errors, raw $P$-values, and Benjamini-Hochberg-adjusted $P$-values from the Mendelian randomization analyses

| **Exposure** | **Outcome** | $\boldsymbol{\beta}$ | **SE** | $\boldsymbol{P}$ | $\boldsymbol{P}_{\boldsymbol{adj}}$ |
| --- | --- | --- | --- | --- | --- |
| **One-sample MR study** | | | | | |
| childhood-onset asthma | IBS | 0.0031 | 0.0010 | 0.0033 | 0.0132 |
| adult-onset asthma | IBS | -0.0021 | 0.0015 | 0.1729 | 0.2767 |
| childhood-onset asthma | IBD | -0.0079 | 0.0030 | 0.0085 | 0.0227 |
| adult-onset asthma | IBD | -0.0014 | 0.0042 | 0.7364 | 0.7364 |
| childhood-onset asthma | PUD | 0.0034 | 0.0015 | 0.0235 | 0.0470 |
| adult-onset asthma | PUD | -0.0023 | 0.0020 | 0.2444 | 0.3258 |
| childhood-onset asthma | GORD | 0.0030 | 0.0009 | 0.0008 | 0.0067 |
| adult-onset asthma | GORD | 0.0012 | 0.0015 | 0.4407 | 0.5037 |
| **Two-sample MR study** | | | | | |
| childhood-onset asthma | IBD | -0.0076 | 0.0033 | 0.0207 | 0.0620 |
| adult-onset asthma | IBD | -0.0069 | 0.0045 | 0.1283 | 0.2566 |
| childhood-onset asthma | CD | -0.0050 | 0.0044 | 0.2573 | 0.3258 |
| adult-onset asthma | CD | -0.0016 | 0.0053 | 0.7610 | 0.7610 |
| childhood-onset asthma | UC | -0.0104 | 0.0040 | 0.0099 | 0.0593 |
| adult-onset asthma | UC | -0.0064 | 0.0058 | 0.2715 | 0.3258 |

**Table S10** Results of the MR-PRESSO global test for detecting horizontal pleiotropy and between SNP-heterogeneity based on the one-sample radial Mendelian randomization framework

| **Exposure (Asthma)** | **Outcome** | **PRESSO RSSobs** | $\boldsymbol{P}_{\boldsymbol{RSSobs}}$ | **Cochran´s Q** | $\boldsymbol{P}_{\boldsymbol{Q}}$ |
| --- | --- | --- | --- | --- | --- |
| childhood-onset | IBS | 74.848 | 0.798 | 73.149 | 0.795 |
| adult-onset | IBS | 39.266 | 0.592 | 37.357 | 0.590 |
| childhood-onset | IBD | 112.748 | 0.002 | 109.641 | 0.002 |
| adult-onset | IBD | 50.514 | 0.039 | 47.641 | 0.037 |
| childhood-onset | PUD | 96.53 | 0.223 | 94.507 | 0.225 |
| adult-onset | PUD | 38.959 | 0.550 | 37.017 | 0.561 |
| childhood-onset | GORD | 99.556 | 0.120 | 97.176 | 0.121 |
| adult-onset | GORD | 54.365 | 0.041 | 51.044 | 0.039 |

**Table S11** Results of the MR-PRESSO global and MR-Egger intercept tests for detecting horizontal and directional pleiotropy, respectively, and between SNP-heterogeneity based on the two-sample radial Mendelian randomization framework

| **Exposure (Asthma)** |  | **Outcome** | **PRESSO RSSobs** | $\boldsymbol{P}_{\boldsymbol{RSSobs}}$ | **Radial**  **Egger intercept** | $\boldsymbol{P}_{\boldsymbol{\beta}_{\boldsymbol{0}}}$ | **Cochran´s Q** | $\boldsymbol{P}_{\boldsymbol{Q}}$ |
| --- | --- | --- | --- | --- | --- | --- | --- | --- |
| childhood-onset |  | IBD | 94.359 | 0.002 | -0.017 | 0.987 | 91.002 | 0.002 |
| adult-onset |  | IBD | 40.910 | 0.065 | 0.899 | 0.510 | 37.906 | 0.062 |
| childhood-onset |  | CD | 105.885 | 0.001 | -1.743 | 0.117 | 102.444 | 3E-04 |
| adult-onset |  | CD | 36.832 | 0.195 | -0.330 | 0.777 | 34.334 | 0.190 |
| childhood-onset |  | UC | 113.504 | 0.001 | 0.297 | 0.725 | 109.915 | 2E-05 |
| adult-onset |  | UC | 46.608 | 0.022 | 0.369 | 0.798 | 43.184 | 0.025 |
